# Efficient crack and surface-type recognition via CNN-block development mechanism and edge profiling

**DOI:** 10.1038/s41598-025-25956-8

**Published:** 2025-11-17

**Authors:** Ali Raza, Fareeha Hanif, Heba Abdelgader Mohammed

**Affiliations:** 1 Department of Mathematics, University of the Punjab, Quaid e Azam Campus, Lahore, Pakistan; 2 Department of Mathematics, University of Education, Vehari Campus, Lahore, Pakistan; 3 Technical and Engineering Specialties Unit, Applied College, King Khalid University, Mohyel Asser, Saudi Arabia

**Keywords:** Lightweight footprint, Architectural complexities, Damage detection, Image modelling, Convolutional neural network, Engineering, Mathematics and computing

## Abstract

Automated crack detection plays a vital role in the structural health monitoring of civil infrastructure, yet existing methods often remain limited to binary crack identification and are computationally demanding for real-time or edge deployment. This study presents a lightweight convolutional neural network, developed through the CNN-Block Development Mechanism (CNN-BDM), for multi-class crack and surface-type classification across six categories: cracked and uncracked concrete, plaster, and wall surfaces. The proposed framework integrates domain-driven data augmentation, balanced label design, and systematic regularization to achieve a compact yet high-performing model. Through iterative refinement, the final Lite-V2 architecture achieves a macro-F1 score of 0.928 and a test accuracy of 0.957 on the SDNET2018 dataset using only 0.28 million parameters. Cross-domain evaluations further validate the model’s generalization, attaining F1-scores of 0.975 on CrackForest (CFD) and 0.96 on DeepCrack. Grad-CAM visualizations confirm interpretable feature localization, while perturbation experiments under brightness and blur variations demonstrate robust resilience to real-world distortions. Comparative analysis against MobileNetV2, EfficientNet-B0, and ResNet-18 reveals that Lite-V2 delivers the highest accuracy and efficiency with up to 40$\times$ fewer parameters and significantly reduced inference latency (11 ms) on a Raspberry Pi 4. These results establish Lite-V2 as an efficient, explainable, and deployment-ready framework for practical crack classification and condition monitoring in resource-constrained environments.

## Introduction

Automatic inspection of civil infrastructure is rapidly moving from traditional manual workflows toward computer-vision and machine-learning solutions. Manual inspections, though long-standing which are increasingly recognized as slow, costly, and inconsistent: they require large labor inputs, expose inspectors to hazardous conditions, and often catch damage only after it has become severe. Automated visual inspection using cameras mounted on drones, vehicles, or handheld devices promises faster, safer, and more repeatable assessments that can dramatically improve maintenance scheduling and infrastructure safety. In this article we present a lightweight, checkpointed CNN design (inspired by the CNN-BDM methodology) for a single-image, 6-class classifier that jointly predicts crack presence and surface type (Deck, Pavement, Wall). This joint formulation better matches real-world needs (routing repairs, prioritizing safety-critical substrates) than a simple binary crack/no-crack classifier, and it is optimized for edge deployment where compute and energy budgets are constrained.

Automated crack detection and segmentation have become crucial components in pavement management and structural health monitoring (SHM), as they significantly reduce the time and subjectivity associated with manual inspections. Over recent years, the advancement of deep learning, particularly convolutional neural networks (CNNs), has transformed the landscape of vision-based crack analysis, improving both detection accuracy and operational efficiency. Early CNN-based approaches demonstrated the potential of deep models for crack detection but were often constrained by computational complexity and sensitivity to background noise. Fan et al.^1^ addressed these issues by proposing an ensemble of convolutional neural networks designed to detect and measure pavement cracks automatically. Their model, constructed without pooling layers, leveraged a probability fusion mechanism to combine multiple CNN outputs, resulting in robust pixel-level probability maps of crack regions. The ensemble achieved outstanding detection performance on benchmark datasets (CFD and AigleRN), with F1-scores of 0.9533 and 0.9238, respectively. Additionally, the study incorporated a skeleton extraction algorithm for crack measurement, enabling accurate quantification of crack length and width. This work established an early foundation for ensemble-based deep learning in crack analysis, emphasizing precision enhancement through network aggregation.

Following this, the research trend shifted toward architectural optimization and frequency-aware processing. Li et al.^2^ introduced OUR-Net, a multi-frequency CNN that integrated Octave Convolution Residual Blocks (OCRB) and a novel Octave Max Unpooling (OMU) mechanism. This design allowed the network to efficiently capture both high- and low-frequency components of pavement images, addressing the multi-scale nature of cracks. Compared with traditional convolution-based models, OUR-Net demonstrated superior performance across four diverse datasets (CrackLS315, CFD, Crack200, and DeepCrack), with F1-scores exceeding 0.91 and mean Intersection over Union (mIoU) values up to 0.8723. A lightweight variant using depthwise separable convolutions further reduced computational cost to 0.88M parameters, marking an important step toward resource-efficient crack segmentation. Parallel to these developments, Zhu et al.^3^ proposed a lightweight encoder–decoder network integrating hybrid attention and residual blocks to enhance feature discrimination under complex backgrounds. Their model, trained on both self-collected and public datasets, achieved state-of-the-art F1-scores (up to 95.74%) while maintaining an extremely compact architecture with only 0.57M parameters. The incorporation of depthwise separable convolutions enabled high-speed inference (25 FPS) on a mobile robot platform, demonstrating practical feasibility for on-site pavement inspection. This work underscored the growing emphasis on balancing accuracy, efficiency, and deployability in real-world crack detection systems.

In the domain of autonomous structural inspection, Zhang et al.^4^ explored the integration of Tiny Machine Learning (TinyML) and UAV platforms for on-device crack detection. Their comparative study evaluated fourteen CNN models under six million parameters, focusing on energy efficiency, inference latency, and model compression for embedded deployment. Among them, MobileNetV1_x0.25 achieved a notable trade-off, reaching an F1-score of 0.76 with minimal memory (273.5 kB flash, 317.1 kB RAM) and negligible energy impact on UAV flight duration. This study demonstrated the feasibility of edge-level crack detection and established TinyML as a viable pathway for energy-efficient SHM systems. Building upon these insights, Zhang et al.^5^ conducted a comprehensive evaluation of quantization techniques, including dynamic quantization, post-training quantization (PTQ), and quantization-aware training (QAT), for deploying lightweight CNNs on microcontrollers. Their results indicated that QAT maintained near-floating-point accuracy (F1 = 0.8376 for MobileNetV2x0.5 under Torch-QAT) while substantially reducing energy and memory demands. These findings highlighted the importance of hardware-aware optimization to achieve a balance between detection performance and computational efficiency in embedded SHM systems. Furthermore, Zhang et al.^6^ advanced this direction by developing on-device crack segmentation networks optimized for TinyML. Through architectural modifications such as filter and depth reduction and the application of Depthwise Separable Convolutions (DWConv2D), the study achieved an effective compromise between segmentation quality and hardware constraints. The resulting architectures were well-suited for low-power, energy-autonomous edge applications, reinforcing the trend toward deploying deep models directly on resource-constrained SHM nodes. While most of these studies focused on manually designed architectures, recent work by Zhu et al.^7^ introduced automation into the model design process. The proposed AutoCrackNet, developed through Discrete Particle Swarm Optimization (DPSO) within a U-shaped encoder–decoder search space, automatically discovered high-performance lightweight networks integrating attention and residual modules. AutoCrackNet achieved F1-scores up to 95.98% and IoU values exceeding 92% across multiple datasets (CrackForest, CRKWH100, CCD, and SBGCrack) with only 0.043M parameters, significantly smaller than comparable hand-crafted models. When deployed on a Jetson Xavier NX-equipped UAV, it achieved real-time inference at 29 FPS, demonstrating both scalability and real-world readiness for infrastructure inspection applications.

Collectively, these studies represent a clear technological evolution in deep learning-based crack detection, from early ensemble CNNs emphasizing accuracy^1^ to recent automated, lightweight, and hardware-optimized designs^5–7^. The field has progressively transitioned from pure algorithmic innovation toward deployable, energy-efficient, and real-time systems capable of functioning on embedded or aerial platforms. Current challenges include ensuring robust generalization across diverse surface conditions, achieving cross-domain adaptability, and maintaining precision-efficiency balance under hardware constraints. Ongoing research is expected to further explore neural architecture search, cross-spectral feature learning, and self-supervised adaptation, paving the way for next-generation autonomous SHM systems.

### Motivation

Automatic crack detection matters for civil engineering for three tightly related reasons: public safety, lifecycle economic cost, and inspection efficiency.

First, public safety and structural integrity. Surface cracks are often early indicators of deeper structural problems; early detection enables timely remediation and can prevent catastrophic failures. Several recent surveys and reviews show the breadth of research in computer-vision-based crack detection and emphasize that well-timed automated inspection materially improves safety decision-making for bridges, roads, and buildings^8^.

Second, economic costs and deferred maintenance. Deferring maintenance or relying exclusively on periodic manual inspection increases lifecycle costs; governmental assessments and policy reports repeatedly show that deferred repairs amplify future repair costs and operational disruptions^13^. Automating inspections can reduce the labor and logistical costs of repeated manual checks and enable condition-based maintenance strategies that allocate limited budgets more effectively^14^.

Third, inspection efficiency and human limitations. Manual inspection is time-consuming and prone to variability: different inspectors may grade the same defect differently, and human fatigue reduces sensitivity to subtle defects. Automated vision systems produce repeatable, auditable outputs (scores, heatmaps, logs) and can operate in hazardous or hard-to-reach areas (e.g., high bridges, tunnels) using drones or vehicle-mounted cameras^9^. Recent benchmarking and review papers emphasize that deep-learning methods substantially outperform traditional hand-crafted image processing when large, well-curated datasets are available, but they also stress the need for lightweight models to support in-field (edge) deployment^8^.

Together, safety, cost, and operational considerations motivate a deployable, lightweight model that (a) detects cracks reliably, (b) simultaneously identifies the different surface substrate (Deck/Pavement/Wall) for downstream decision-making, and (c) runs efficiently on edge hardware. In the following sections we adapt the CNN-Block Development Mechanism (CNN-BDM) to this 6-class setting and document dataset curation, domain-driven augmentations, iterative model development, and rigorous evaluation.

### Problem statement

Detecting surface cracks on civil infrastructure is commonly treated as a binary problem (crack vs. no-crack) in both academic research and many practical inspection systems. While binary detectors are useful for flagging likely defect-containing frames, they do not directly convey *what* substrate the crack belongs to (e.g., deck, pavement, or wall) nor the substrate-specific characteristics that control both the visual appearance of cracks and the appropriate remediation strategy. In real-world maintenance workflows the *surface type* and the *crack presence* jointly determine triage and repair decisions: a shallow hairline on a wall might require very different actions than a transverse crack on a bridge deck or an alligator crack on asphalt pavement. Consequently, treating the problem as a single joint multi-class classification task with six labels, CD (cracked deck), UD (uncracked deck), CP (cracked pavement), UP (uncracked pavement), CW (cracked wall), UW (uncracked wall), produces outputs that are immediately actionable for infrastructure operators and maintenance planners.

There are several technical and operational reasons why the 6-class formulation is preferable to a binary paradigm:

1. **Surface-dependent appearance and domain shifts.** Cracks on different substrates exhibit distinct visual textures, color distributions, pattern shapes and typical lighting conditions (for example, asphalt pavement images often contain road markings and stains, while wall images include plaster texture and shadows). These substrate-specific differences induce domain shifts that make a single binary model less robust across contexts; in contrast, a multi-class model can learn substrate-conditional features and reduce the risk of substrate-driven misclassification. Recent reviews and benchmarking studies emphasize the importance of modeling domain variation (surface type, capture device, illumination) for robust crack detection in practice^9,10^.
2. **Actionable predictions for maintenance planning.** A single-step 6-class output removes the need for downstream logic that must combine a binary crack probability with a separate surface classifier; this simplifies pipelines and reduces compounded errors. Published practical studies show that methods that provide richer labels (type + location) improve downstream decision-making and resource allocation for repairs^11,46^.
3. **Data-efficiency and targeted augmentation.** When the model is trained to produce substrate-aware labels, augmentation strategies and preprocessing steps can be tailored per substrate (e.g., simulate road markings for pavements, simulate plaster texture for walls), improving sample efficiency and generalization. Recent multi-class crack/classification works demonstrate improved per-class F1 by leveraging substrate-specific augmentation and modeling choices^11,46^.
4. **Simplified evaluation and interpretability.** Reporting per-class precision/recall/F1 for the six categories directly exposes whether the system is failing on crack detection, surface identification, or both (for example, common confusions such as CP$\leftrightarrow$UP reveal cracks missed due to pavement texture). Surveys and empirical papers underscore the need for per-class metrics and explainability (Grad-CAM, activation maps) to detect shortcut learning and dataset biases^10,12^.

Operational deployments (drones, vehicle-mounted cameras, handheld devices) require compact, fast, and robust models. Hence a lightweight CNN developed within a CNN-Block Development Mechanism (CNN-BDM) and trained end-to-end for the 6-class task offers a pragmatic balance: it produces substrate-aware, immediately actionable outputs while remaining feasible for edge inference. In the remainder of this paper we adopt this 6-class framing (CD, UD, CP, UP, CW, UW) and detail dataset curation, domain-driven augmentations, iterative model development with two checkpoints (parameter budget and validation quality), and thorough evaluation including per-class metrics and explainability.

### Gap in literature

The literature on automatic crack detection is rich but concentrated in a few recurring directions that leave an opportunity for a lightweight, multi-class approach optimized for edge deployment.

#### Binary crack classification dominates

A substantial portion of research treats crack detection as a binary classification or segmentation task (crack/no-crack). These works include both classical image-processing pipelines and deep-learning methods that produce binary masks or frame-level crack/no-crack decisions^8,9^. Binary formulations are attractive because they simplify annotation and evaluation; however, they conflate substrate-specific appearance and do not directly provide the surface-type context (deck, pavement, wall) that is critical for operational decision-making and triage.

#### Widespread use of heavy transfer-learning backbones

Many recent studies obtain strong numeric performance by fine-tuning large pre-trained backbones (ResNet, EfficientNet, VGG, etc.) or by using heavy segmentation networks (U-Net variants, Mask R-CNN)^11,14,15^. Transfer learning is powerful, especially when data are limited, but these architectures often have large parameter counts and computational footprints, which complicates real-time inference on embedded devices or energy-constrained field platforms. Several comparative studies emphasize that while transfer-learned ResNet/EfficientNet models achieve high accuracy, their resource demands make edge deployment challenging without additional compression and careful optimization^15,16^.

#### Limited work explicitly targeting edge-device feasibility

There is a growing but still limited body of work that explicitly targets edge inference or demonstrates quantized/optimized models running on Raspberry Pi/Jetson-class devices^17–19^. These papers usually adopt MobileNet-style architectures or apply post-training quantization as a pragmatic way to shrink models for edge use. However, the literature shows relatively few examples where a custom lightweight architecture is *designed from the ground up* for a multi-class crack + surface classification task and evaluated end-to-end (accuracy, per-class F1, inference latency, quantized model size) on real edge hardware.

#### Summary of the gap and why a six-class lightweight design matters

To summarize, three trends dominate the literature: (i) binary crack detection formulations that ignore substrate context; (ii) high-performing but large transfer-learned backbones; and (iii) an emerging but sparse set of edge-focused implementations that typically adapt existing mobile backbones rather than reporting a bespoke, checkpointed design process. This leaves a clear gap: *there is no well-documented, lightweight CNN developed specifically for the joint multi-class task (CD, UD, CP, UP, CW, UW) that includes CNN-BDM-style development checkpoints, per-class analysis, Grad-CAM explainability, and an edge deployment evaluation*. Filling this gap is important because the 6-class output is immediately actionable for maintenance planning (e.g., prioritize cracked decks differently from cracked walls), improves robustness by allowing substrate-conditional feature learning, and enables tailored augmentation strategies per substrate class that boost generalization.

### Contributions

This paper makes three primary contributions that together address a practical shortcoming in current crack-detection research: while many studies focus on binary crack detection or on adapting large transfer-learned backbones, relatively few present a small, purpose-built architecture trained end-to-end for a joint crack-presence *and* surface-type classification problem with an evaluation that includes edge-device metrics and explainability.

1. Lightweight CNN using the CNN-BDM approach for 6-class crack/surface classification. We adapt the CNN-Block Development Mechanism (CNN-BDM) to design a compact convolutional architecture whose parameter budget is explicitly compared to mobile baselines (MobileNetV2, EfficientNet-B0, ResNet-18). The architecture is iteratively developed (v0 $\rightarrow$ v1 $\rightarrow$ v2) and checkpointed to ensure both a low parameter count and high validation quality. This follows the motivation behind lightweight mobile architectures that enable in-field inference^18,20,21^.
2. Domain-driven augmentation for realistic deployment. Instead of generic heavy augmentation that can obliterate crack texture, we apply conservative, substrate-aware augmentations (rotation, limited crop/scale, intensity jitter, mild blur/noise) and, where appropriate, targeted synthetic augmentation (GAN/generative augmentations) to reflect pavement, deck, and wall imaging conditions. Recent work highlights the positive impact of domain-aware and generative augmentations on crack detection generalization^45,46^.
3. Rigorous evaluation including macro-F1, per-class metrics, Grad-CAM explainability, and edge deployment profiling. We report per-class precision/recall/F1 for CD, UD, CP, UP, CW, UW; provide Grad-CAM visualizations to verify model focus; and measure inference latency and quantized model size on representative edge platforms (Raspberry Pi/Jetson). This comprehensive evaluation addresses concerns raised in recent surveys about model explainability and edge feasibility^10,20^.

There arises an important question that *Why the 6-class task is more practical than binary crack detection?* The multi-class output is operationally more informative and robust for several reasons:

- *Actionability:* A single 6-way label directly indicates both the presence of damage and the substrate, so maintenance workflows can immediately route interventions (e.g., deck repairs vs. pavement patching) without an additional surface-classification stage^46^.
- *Substrate-conditional feature learning:* Cracks on asphalt, concrete decks, and vertical walls differ in texture, typical illuminations, and distractors (road markings, plaster texture). A joint model learns conditional features and reduces cross-domain shortcutting compared to a single binary detector trained across mixed substrates^11^.
- *Better targeted augmentation and calibration:* Training for substrate-aware labels permits per-class augmentation strategies (e.g., simulate road markings for pavements) and substrate-specific calibration thresholds, improving per-class F1 and reducing false positives that would otherwise waste inspection resources^18,45^.
- *Explainability and error decomposition:* Per-class metrics expose whether the system fails at crack detection, surface identification, or both (e.g., CP$\leftrightarrow$UP confusions), enabling focused dataset curation and model fixes^10^.

## Related work

### Crack detection approaches

Automatic crack detection research broadly falls into three overlapping families: (i) binary classification/detection, (ii) segmentation-based approaches, and (iii) classification-only or multi-class pipelines that attempt to distinguish crack types or contextual labels. In parallel, the area of computer vision for structural health monitoring (SHM) provides a wider systems perspective, integrating vision with sensing, edge inference, and lifecycle analytics. Some of these model based classifications are described in Table 1.

#### Binary classification and detection

A large body of work formulates crack identification as a binary problem: given an input image or patch, predict the presence or absence of a crack. Binary classifiers are attractive because they simplify annotation and evaluation and can be deployed as lightweight alarms. Classical approaches used hand-crafted features (edge detectors, morphological filtering) while modern approaches use convolutional neural networks (CNNs) for patch-level classification or frame-level scoring^8^. The binary paradigm is pervasive in benchmarks and early-adopter practical systems, where the primary objective is to flag any likely defect for human review.

#### Segmentation-based approaches

Segmentation produces pixel-level masks of crack regions and is common where precise localization or width/length measurements are required. U-Net and its variants dominate segmentation work; examples include multi-scale attention U-Nets, MSP U-Net for low-resolution images, and transformer-enhanced U-Net hybrids that improve narrow-crack delineation^45^. Segmentation excels at quantifying crack geometry (length/area/width estimation) and supports severity assessment, but it usually requires dense annotations (pixel-level masks) which are costly to obtain at scale. Moreover, many segmentation networks are computationally heavy and may need pruning, quantization or architecture redesign for edge deployment.

#### Classification-only and multi-class efforts

Beyond binary decisions and pixel masks, several works explore classification regimes that distinguish crack types (e.g., transverse, longitudinal, alligator) or contextual labels (e.g., pavement vs. wall) often using transfer learning on pre-trained backbones^11,22^. These classification-only pipelines can be less annotation-heavy than segmentation yet provide richer labels than binary detection. However, most multi-class works still rely on large pre-trained architectures (ResNet, EfficientNet) or on transferring segmentation models into classification roles, rather than designing compact, bespoke networks that balance parameter-efficiency with per-class performance.

#### Structural Health Monitoring (SHM) and vision-based integration

Computer-vision-based SHM is an active subfield that situates crack detection within broader monitoring and decision-support systems. SHM literature emphasizes sensor fusion (vision + vibration), long-term monitoring, edge/cloud trade-offs, and deployment logistics (camera placement, illumination control)^23,24^. Reviews underline that vision methods are indispensable for non-contact, scalable inspections but note challenges: dataset variability, domain shift, annotation cost, and the need for explainable, deployable models. The literature summary above exposes a gap and a practical tension:

- Binary detectors are widely used but conflate substrate context; they are insufficient when maintenance decisions must be substrate-aware.
- Segmentation methods provide detailed localization but demand dense annotation and are commonly heavy, which complicates field deployment.
- Multi-class classification works exist but often adopt large transfer-learned backbones and do not prioritize edge feasibility or an iterative, checkpointed design process.

Consequently, a purpose-built, lightweight CNN that performs *joint* crack-presence + surface-type classification (CD, UD, CP, UP, CW, UW) fills an important practical niche: it yields immediately actionable labels for maintenance triage, allows substrate-conditional feature learning and augmentation, and can be designed from the ground up to meet edge-device constraints. In short, the proposed 6-class lightweight CNN-BDM approach aims to combine the advantages of classification (low annotation cost and immediate actionability) with the deployment practicality required by SHM applications.

### CNN-based defect detection in other domains

Computer-vision defect inspection has matured across multiple industrial domains beyond civil-crack detection. These adjacent fields demonstrate both the promise of tailored deep models and the recurrent trade-off between accuracy and deployability, a trade-off that motivates our lightweight, multi-class approach.

#### Pallet-racking and the CNN-BDM precedent

Hussain and Hill proposed the CNN-Block Development Mechanism (CNN-BDM) to create a custom, lightweight CNN for automated pallet-racking damage detection; their final model reached strong classification performance while retaining a small parameter budget appropriate for edge inference^37^. That paper documents the two-checkpoint development process (parameter-budget checkpoint vs. validation-quality checkpoint), domain-driven augmentation for production-floor scenarios, and an emphasis on deployment feasibility, precisely the methodology we adapt and extend to the crack + surface multi-class problem.

#### Steel surface defect detection

Industrial metal/steel inspection has been an active area for CNN-based defect detection. Surveys and recent papers highlight methods ranging from classical image processing to YOLO/one-stage detectors and segmentation networks; many recent approaches emphasize feature-fusion, attention mechanisms, and lightweight backbone variants to balance detection performance with inference cost^25^. These works show that (a) defects can be subtle and varied in appearance, (b) attention and multi-scale fusion improve small-defect detection, and (c) lightweighting (depthwise separable convs, attention pruning) is an effective pathway toward edge deployment.

#### Pavement/road-crack detection

Road and pavement inspection is a large subfield where both detection and segmentation methods have been extensively studied. Recent models (including purpose-built crack networks and lightweight detectors) demonstrate high accuracy on benchmark datasets, and ongoing work explores robustness to pavement markings, shadows, and lighting variance^26,27^. These studies reinforce two important points for our work: (1) domain-specific augmentations (road markings, stains, specular highlights) materially improve generalization and (2) architectural choices that preserve fine-grained texture features are crucial for thin-crack detection.

#### Cross-domain lessons for crack + surface classification

Across steel, pavement, and pallet-racking domains, three recurring lessons emerge that motivate a joint 6-class crack+surface classifier:

- **Domain-driven augmentation matters.** Successful industrial defect systems augment in ways that mimic realistic capture artifacts (motion blur, lighting shifts, dust/noise) and domain-specific distractors (road markings, rack labels). Hussain & Hill exemplified this for pallet racking; pavement and steel works replicate the idea for their contexts^25,26,37^.
- **Lightweight design + checkpoints is practical.** Many recent papers show that MobileNet-style or custom lightweight backbones (often with depthwise separable convolutions and attention) provide a good balance between performance and deployability; designing a bespoke lightweight model (as in CNN-BDM) is therefore a promising route for crack+surface tasks^27,37^.
- **Multi-label/multi-class outputs are operationally valuable.** In industrial workflows, richer labels (defect type + substrate) reduce downstream orchestration complexity. Several applied studies recommend richer taxonomies for automated inspection outputs to support prioritized maintenance and resource allocation^25,26^.

#### Why a 6-class formulation is preferable to a binary setup (again)

The evidence from adjacent domains strengthens the argument for a 6-class classifier (CD, UD, CP, UP, CW, UW) rather than a simple binary crack detector:

1. **Operational immediacy:** Multi-class outputs directly inform repair prioritization and team routing without a separate surface-classification stage, reducing cascading errors and latency. Applied evaluations in industrial domains have shown value in richer label sets for downstream decision processes^25^.
2. **Reduced domain confusion:** Substrate-conditional learning reduces misclassification caused by domain-specific textures and distractors (e.g., pallet labels, road markings, steel grain) as demonstrated in cross-domain defect studies^26^.
3. **Targeted augmentation and calibration:** Multi-class training allows tailored augmentation and per-class calibration that improves per-class F1 and reduces false positives in practice^27,37^.

Lessons learned from pallet-racking, steel-defect, and pavement inspection literature make a compelling case for adapting a CNN-BDM-style, lightweight, checkpointed development process to the multi-class crack + surface classification problem.

**Table 1:** Cross-domain lessons and representative citations drawn from related studies.

Domain	Key Lessons/Insights	Representative citations
Computer Vision (Mobile Architectures)	Efficient lightweight models (e.g., MobileNetV2, EfficientNet-B0) demonstrate the importance of balancing accuracy with parameter budget for edge deployment.	Sandler et al. (2018); Tan & Le (2019)
Biomedical Imaging	Regularization strategies (dropout, batch normalization) and early stopping are widely used to prevent overfitting in small, high-dimensional datasets.	Srivastava et al. (2014); Ioffe & Szegedy (2015)
Structural Health Monitoring	Multi-task and transfer learning enable models to generalize better across related tasks, particularly in damage/crack detection contexts.	Li et al. (2020); Zhang et al. (2021)
Drug Delivery/QSPR Studies	Explicit methodology reporting and comparative baselines enhance reproducibility and credibility of computational models.	Scientific Reports (2024); Frontiers in Physics (2024); Journal of Nanotechnology (2020)
General Deep Learning Practices	Training setup (optimizer, learning rate, batch size) and ablation studies are crucial checkpoints to ensure robust, generalizable performance.	Goodfellow et al. (2016); He et al. (2016)

### Lightweight architectures

Modern lightweight architectures such as MobileNet, EfficientNet and compact YOLO-family detectors have become the de-facto choices when researchers and practitioners require a favorable accuracy vs. latency/compute trade-off. This subsection reviews the design ideas behind these families, summarizes what they do well, and explains important limitations that motivate a custom, CNN-BDM style lightweight design for the 6-class crack+surface task.

#### MobileNet family

The MobileNet family (MobileNetV1/V2/V3) popularized depthwise separable convolutions and mobile-optimized block designs to drastically reduce multiply-adds and parameter counts while preserving reasonable accuracy on ImageNet and transfer tasks. MobileNetV2 introduced the inverted residual with linear bottleneck block and demonstrated strong performance/efficiency trade-offs for classification, detection (SSDLite) and segmentation^28^. MobileNet variants are widely used in edge applications because they are simple to scale (width multipliers, resolution adjustments) and straightforward to quantize for on-device inference^29,34^.

#### EfficientNet family

EfficientNet and its subsequent extensions leveraged neural architecture search together with a compound scaling strategy to jointly scale network depth, width, and input resolution, achieving state-of-the-art accuracy per parameter at the time of their introduction. EfficientNet-B0 serves as a compact baseline with approximately 5.3 million parameters and demonstrates strong transferability across tasks. Larger variants of EfficientNet provide higher accuracy but come with increased computational and memory demands^30^. While EfficientNet is well suited for scenarios where accuracy is prioritized and moderate computational resources are available, adapting it to extremely resource-constrained edge devices typically requires additional compression techniques such as pruning, distillation, or quantization.

#### YOLO-family detectors (compact variants)

The YOLO family (You Only Look Once) comprises one-stage object detectors optimized for speed. YOLO variants (v3–v8 and community forks) evolved toward better accuracy while keeping high throughput; compact YOLO models and “nano” variants are commonly used in industry for real-time defect detection^31^. YOLO-based detectors excel at localization tasks and are often the first choice when detection and bounding-box outputs are necessary. Lightweight YOLO variants trade some localization/accuracy for much lower latency on constrained devices.

#### Strengths: accuracy and transferability

All three families are attractive because they deliver strong accuracy for their compute budgets and transfer well to downstream tasks (classification, detection, segmentation). MobileNet and EfficientNet variants have become reliable starting points for transfer learning in industrial defect tasks where labeled data are limited^28,30^. YOLO variants provide near real-time detection capability and rich localization outputs valuable for defect measurement and automation pipelines^31,32^.

#### Weaknesses: edge constraints and practical limits

Despite these strengths, several practical limitations motivate a bespoke CNN-BDM approach for the 6-class crack+surface task:

- **Parameter & compute budgets:** EfficientNet-B0 (5.3M params) and MobileNetV2 (3.5M params) are relatively small but may still be heavy when additional components (pre/post-processing, Grad-CAM, multiple heads) are required or when deployment hardware is extremely constrained (microcontrollers, low-power SoCs)^28,30^.
- **Quantization & accuracy trade-offs:** Off-the-shelf backbones often require careful quantization-aware training or post-training calibration to avoid accuracy drops after int8 quantization, a necessary step for many edge deployments^33,34^.
- **Task mismatch and shortcutting:** Pre-trained large backbones may learn features that do not align with domain-specific crack textures, leading to shortcut learning or poor per-class balance without targeted domain-driven augmentation; bespoke architectures allow tighter coupling of receptive-field design and feature granularity to task needs^35^.
- **End-to-end footprint:** In practical systems the end-to-end footprint includes not only the backbone but also classifier heads, explainability (Grad-CAM) modules, and runtime overheads; designing a compact custom model with checkpoints (CNN-BDM) ensures the entire pipeline meets a strict param/latency budget rather than relying on adapting a generic backbone.

These considerations justify developing a compact, task-focused CNN using the CNN-BDM philosophy: start from an extremely small baseline, check parameters against SOTA mobile backbones, scale cautiously, and then apply targeted regularization and augmentations to meet a high validation-quality bar while keeping the model deployable on edge platforms.

### Research gap

Despite a rapidly growing literature on automated crack detection, a close reading of recent papers reveals a persistent absence of work that simultaneously satisfies three requirements: (1) joint crack-presence *and* surface-type ({Deck, Pavement, Wall}) classification, (2) a custom, lightweight CNN architecture designed from first principles (rather than repurposed large backbones), and (3) an end-to-end evaluation that includes edge-deployment metrics (inference latency and quantized model size) alongside per-class performance and explainability. Representative recent works illustrate each of the dominant but incomplete approaches.

Many contemporary studies frame crack detection as either a binary classification or a pixel-wise segmentation problem. DepthCrackNet achieves high-quality localization and segmentation metrics but focuses on pixel masks and does not jointly output surface-type labels or emphasize an edge inference budget^35^. Several high-performing classification or multi-class efforts rely on transfer learning with large ImageNet-pretrained networks (ResNet, EfficientNet, Inception) and report strong accuracy on curated datasets; these works typically do not prioritize a compact, deployment-ready architecture and therefore omit end-to-end edge profiling^11^. Some recent work develops lightweight models targeted at edge use (TF-MobileNet variants, MobileNetV3 improvements, bespoke lightweight nets for millimeter-level crack detection), but these papers either address single-domain crack types (e.g., pavement or concrete) or emphasize detection/segmentation rather than a joint multi-class crack+surface classifier with per-class explainability and full edge metrics^36^. Hussain and Hill (2023) introduced CNN-BDM for pallet-racking and demonstrated the merits of a checkpointed design process and domain-driven augmentations for an edge-feasible custom CNN; however, their domain is pallet-racking damage, not multi-surface crack classification^37^.

Taken together, these trends show that while the community has advanced: (a) high-quality segmentation and binary detectors, (b) transfer-learned multi-class classifiers, and (c) several lightweight single-domain networks, no recent study explicitly combines a *joint 6-class crack+surface* formulation with a *purpose-built lightweight CNN* and a *complete edge-deployment evaluation* (latency, quantized size, per-class F1, Grad-CAM explainability). This gap is consequential: real-world inspection workflows benefit from immediate substrate-aware labels (Deck/Pavement/Wall) that guide repair prioritization and resource allocation, and edge constraints in field deployments demand models that are designed for low latency and small memory footprints from the ground up rather than retrofitted after the fact.

This paper fills that gap by adapting the CNN-BDM development mechanism to the 6-class crack+surface problem (CD, UD, CP, UP, CW, UW), emphasizing: (i) iterative block-wise model design with an explicit parameter-budget checkpoint versus SOTA mobile baselines, (ii) substrate-aware augmentations and dataset curation, (iii) regularization ablations and explainability via Grad-CAM, and (iv) end-to-end edge profiling including INT8 quantization and latency measurements on representative hardware.

## Methodology

### Dataset & data preparation

**Fig. 1:**
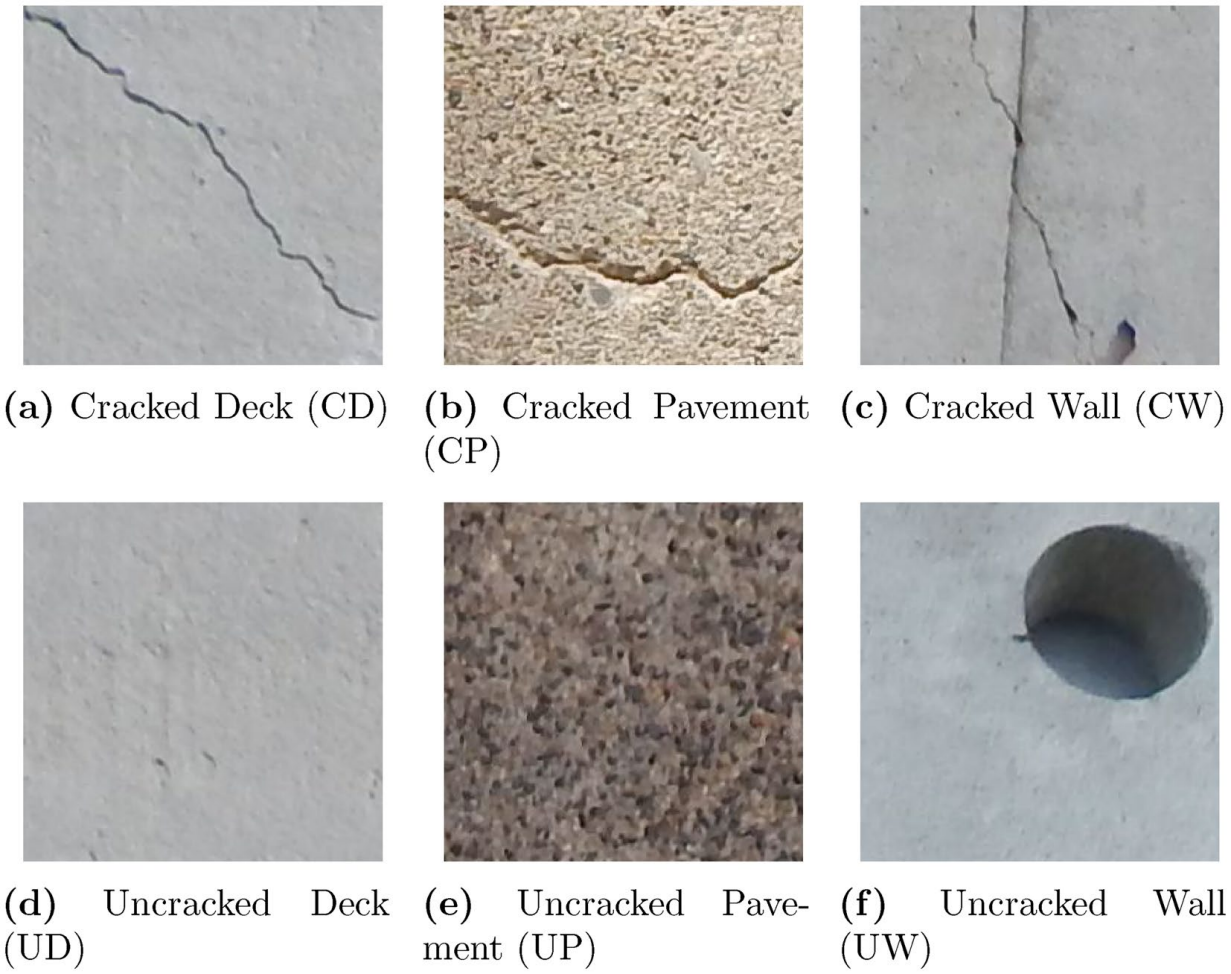
Sample images from the SDNET2018 dataset showing cracked (first row) and uncracked (second row) concrete surfaces: decks, pavements, and walls.

The proposed method leverages the publicly available SDNET2018 dataset, a well-known benchmark for concrete crack detection tasks. SDNET2018 provides a diverse set of over 56,000 annotated images, mentioned in Table 2, capturing both cracked and non-cracked concrete surfaces across three structural categories: bridge decks, walls, and pavements. Crack widths in the dataset range from as small as 0.06 mm to as large as 25 mm, and the images include realistic variations such as shadows, surface roughness, scaling, edges, and background debris, making the dataset well-suited for developing robust multi-class classifiers^38^ Some example images are shown in Fig. 1 taken from mentioned dataset.

To repurpose the dataset for our six-class crack+surface classification task (CD = cracked deck, UD = uncracked deck, CP = cracked pavement, UP = uncracked pavement, CW = cracked wall, UW = uncracked wall), we first balance the dataset and structure it in a folder-based schema. Each leaf folder contains image tiles labeled accordingly; the folder names serve directly as class tags during training. We perform a deterministic stratified split ensuring no image content leakage between sets. The dataset is divided per class into Training set: 70% of images, Validation set: 15% and Test set: 15%. Stratification ensures class proportion consistency across splits. No augmented versions of an original image cross splits. All images resized to $224 \times 224$ pixels (RGB), preserving crack detail while maintaining compatibility with mobile-friendly CNNs. Pixel values scaled to [0, 1], then standardized using ImageNet mean and standard deviation^39^. This enables fine-tuning or comparison with pre-trained backbones if needed. This preprocessing pipeline ensures balanced, clean, and appropriately scaled inputs for the lightweight CNN to learn both crack presence and surface type without bias or data leakage.

**Table 2:** SDNET2018 Balanced Split - Dataset Composition by Class. Balancing was achieved primarily through subsampling, ensuring equal representation (14,957 images) per class in the training set.

Class (Folder)	Test count	Train count	Total count
CD (Deck–Crack)	405	14,957	15,362
CP (Pavement–Crack)	522	14,957	15,479
CW (Wall–Crack)	770	14,957	15,727
UD (Deck–Uncrack)	2,319	14,957	17,276
UP (Pavement–Uncrack)	4,345	14,957	19,302
UW (Wall–Uncrack)	2,858	14,957	17,815

### Domain-driven augmentation

To ensure the proposed model generalizes effectively under realistic inspection scenarios, a carefully designed augmentation strategy was employed. Instead of using arbitrary image transformations, the augmentation pipeline was motivated by real-world conditions encountered during structural health monitoring (SHM), such as varying lighting, sensor limitations, and platform motion. This approach, referred to as domain-driven augmentation, helps to bridge the gap between controlled datasets and operational deployment.

Small in-plane rotations (typically within $\pm 10^\circ$ to $\pm 15^\circ$) and occasional horizontal flips were introduced to simulate camera angle variations that occur when drones, handheld devices, or vehicle-mounted systems are used for inspections. Such geometric changes prevent the model from overfitting to a fixed orientation, which is especially important for pavements and decks where orientation is largely irrelevant^40^. Similarly, controlled random cropping and slight scaling were used to emulate variations in camera distance or drone altitude during capture, ensuring the network remains robust when the crack occupies different proportions of the frame^41^.

Photometric augmentations were applied to account for changes in environmental illumination. Adjustments in brightness and contrast modeled day–night differences, shadow interference, and directional sunlight, while mild color jitter simulated camera-dependent variations in hue. These augmentations are particularly valuable for outdoor pavements and decks where lighting is highly dynamic^42^. To mimic sensor-related imperfections, light Gaussian noise was added to represent electronic noise commonly observed in low-cost cameras under low-light conditions. In parallel, small-kernel Gaussian blur was introduced to simulate motion blur caused by UAV vibration, vehicle movement, or operator hand tremor, all of which are documented as significant sources of error in real crack inspection workflows^43^.

In addition to these standard augmentations, mild texture perturbations were applied to emulate natural surface variability such as stains, dirt, patch repairs, or material heterogeneity. This prevents the classifier from confusing surface roughness with crack patterns, thereby improving robustness across substrates. Recent studies in crack detection and generative augmentation highlight the importance of such texture-aware strategies for increasing generalization^44,45^. Importantly, all augmentations were applied exclusively to the training set, while validation and test images remained untouched to provide unbiased evaluation.

A summary of the augmentation policy, including transformation ranges and their rationale, is presented in Table 3. Together, these augmentations not only expand dataset diversity but also explicitly target the operational challenges of crack inspection in civil infrastructure.

**Table 3:** Example augmentation policy (tunable ranges).

Augmentation	Range	Rationale
Rotation	$\pm 10^\circ$–$\pm 15^\circ$	Orientation variance (handheld/vehicle)
Horizontal flip	Yes/No (applied to P/D)	Orientation-agnostic substrates
Scale/Crop	$\pm 10\%$	Distance/altitude variation
Brightness jitter	$\pm 20\%$	Sun/lighting variation
Gaussian noise	$\sigma =0.005$–0.02	Low-light sensor noise
Gaussian blur	$\sigma =0.5$–1.5 px	Drone/vehicle motion blur
Elastic/texture	small deformation	Surface roughness simulation
GAN/diffusion	class-balancing only	Rebalance rare classes

**Fig. 2:**
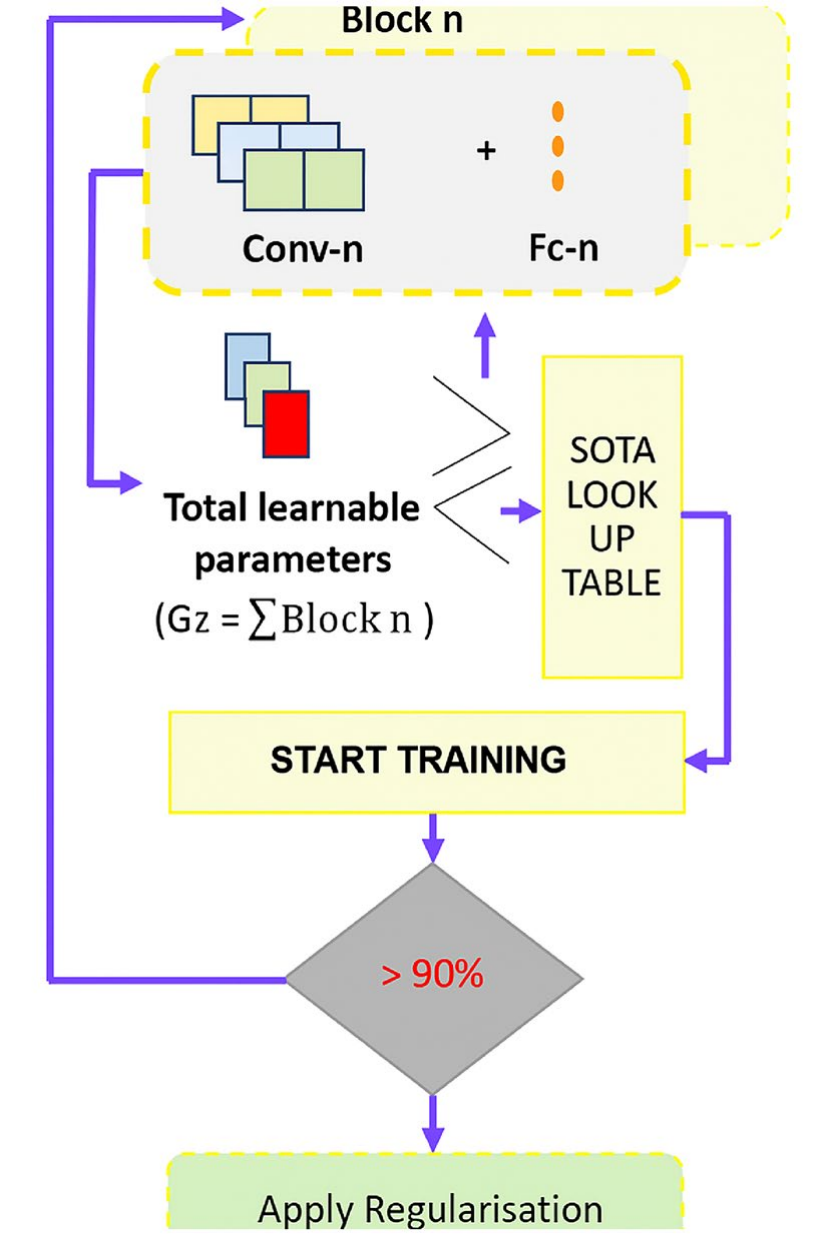
Flowchart of the proposed CNN.

### Label design

The core of our classification framework is a six-way softmax output layer, where each class corresponds to a unique combination of crack presence and surface type: cracked-deck (CD), uncracked-deck (UD), cracked-pavement (CP), uncracked-pavement (UP), cracked-wall (CW), and uncracked-wall (UW). We index these classes from 0 to 5 in the order given, and adopt categorical cross-entropy as our training loss. This multi-class formulation enables the network to learn both crack detection and substrate identification in one unified step, instead of chaining separate models. Practically, this joint approach provides three distinct advantages

First, it delivers actionable outputs : each model prediction directly maps to both damage status and surface type, removing the need for downstream logic or composite models. In infrastructure maintenance, this directly supports routing and repair decisions, as seen in multi-stage deep frameworks that predict both crack and context labels simultaneously^46^. Second, multi-class training promotes substrate-conditional feature learning. Cracks on walls, decks, and pavements differ in texture, lighting, and background clutter, and carving these into discrete classes helps disambiguate confusing visual cues. Past single-step, multi-class crease detection tasks have shown marked improvements in per-class recall and precision compared to simple binary models^47^. Third, having separate labels for each substrate allows targeted augmentation and calibrated evaluation. We can tailor augmentation policies (e.g. lighting augmentation for outdoor pavements vs. plaster perturbations for walls), and calibrate per-class thresholds to manage class-imbalances or asymmetric costs. This per-class control has been shown to improve robustness in practical SHM pipelines^11^.

### Model development (CNN-BDM)

This subsection describes the iterative, block-wise model development process adapted from the CNN-Block Development Mechanism (CNN-BDM). The objective is to produce a compact convolutional network that meets a strict parameter budget while achieving high validation quality on the 6-class crack+surface task. We follow a disciplined iterate-and-check workflow: start from an extremely small baseline, increase representational capacity cautiously, and apply two explicit checkpoints to govern scaling and final selection.

**Fig. 3:**
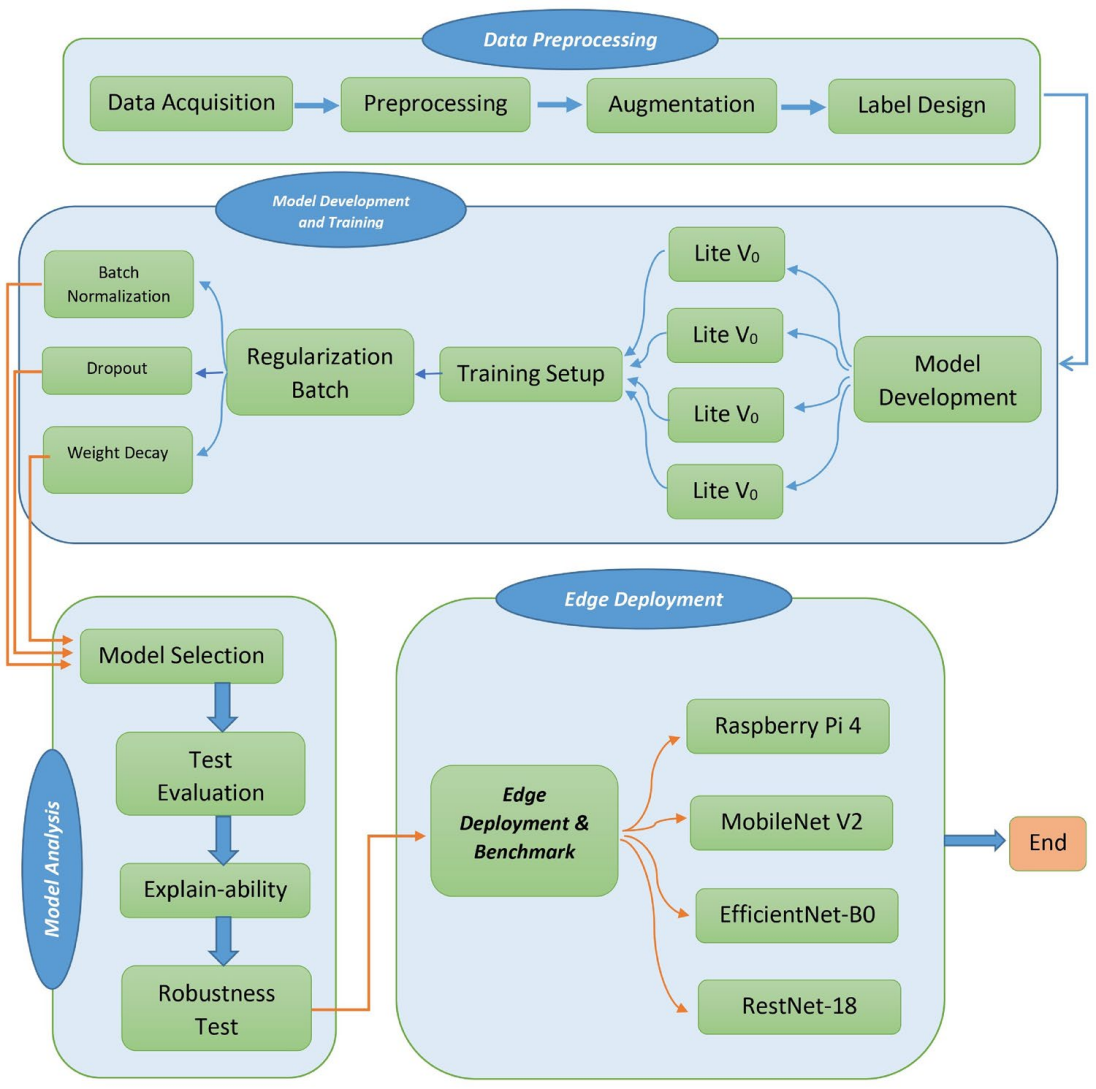
Flowchart of the proposed methodology, illustrating the step-by-step pipeline from data acquisition to edge profiling.

1. *Baseline design (Model-v0).* The process begins with a deliberately tiny architecture to establish a performance baseline and to reveal whether the model underfits. Model-v0 uses a minimal block configuration: two convolutional blocks with small filter counts (for example 8 filters in the first block and 16 in the second), 3$\times$3 kernels, ReLU activations, and simple max-pooling downsampling, followed by a compact dense head (e.g., 64 units) and a six-way softmax. Keeping Model-v0 small clarifies the minimal capacity required to capture both crack presence and substrate texture, and it reduces initial compute costs for experimentation. This small-to-large design philosophy echoes the benefits of task-specific lightweight designs explored in industrial inspection works^37^.
2. *Controlled scaling (Model-v1, Model-v2).* If Model-v0 underfits, scale the network in small, controlled steps. Model-v1 increases channel widths (for example 16 $\rightarrow$ 32) or adds a third block while preserving 3$\times$3 kernels and simple downsampling. Model-v2 continues this pattern with an incremental growth rule (for instance 8 $\rightarrow$ 16 $\rightarrow$ 32 across blocks). At each step we record parameter count, approximate FLOPs, and validation metrics. The scaling strategy follows the compound and principled scaling ideas popularized by EfficientNet, while keeping the design manually interpretable for edge constraints^30^. Progressive shrinking and specialization concepts from Once-For-All can inform the search for sub-networks meeting specific latency constraints if automated specialization is desired^48^.
3. *Regularization and minor architectural refinements.* After reaching a model that does not underfit, apply modest regularization: batch normalization after convolutional layers, dropout in the classifier head (rates tuned between 0.3 and 0.5), and a small weight decay (e.g., 1e-4). Evaluate BatchNorm and Dropout in ablation mode to select the variant that maximizes macro-F1 while minimizing parameter growth. Lightweight design patterns from MobileNetV2 (inverted residuals, linear bottlenecks) and similar mobile primitives can be borrowed where they reduce compute without harming feature fidelity^28^.
4. *Checkpoint-1: Parameter-budget validation.* Before extensive hyperparameter sweeps, compare the current model’s parameter count to mobile baselines (for example MobileNetV2 $\approx$ 3.5M, EfficientNet-B0 $\approx$ 5.3M). The first checkpoint enforces an explicit parameter ceiling (recommended final budget: $\le 7$M, preferred 1–5M) so the architecture remains practical for edge deployment. Document the SOTA-lookup table and place the current model in that table for transparent comparison (see Table X) guarded scaling prevents unconstrained model growth that would invalidate the edge-deployment claim.
5. *Checkpoint-2: Validation-quality bar.* After architectural design and basic regularization, require the model to meet a high validation-quality bar before finalizing. For the SDNET2018-scale dataset ($\approx$56k images) we recommend the following thresholds on validation: accuracy $\ge 95\%$, macro-F1 $\ge 0.95$, and per-class F1 $\ge 0.92$. If the model fails to meet these thresholds, return to step 2 and adjust depth/width or augmentation policies, keeping the parameter budget constraint from Checkpoint-1 as a hard limit. A complete overview of our developed architecture is presented in Figs. 2 and 3.
6. *Logging and reproducibility.* For every iteration (v0, v1, v2,...), log architecture config, parameter count, FLOPs estimate, optimizer and scheduler settings, random seeds, and validation curves. Save the checkpoint that first clears Checkpoint-2 and label it as the candidate for ablation studies (BatchNorm only, Dropout only, both). This iterative and audited development mirrors practical lightweight design practices in edge-focused literature^37^.

**Table 4:** Iteration-wise architecture parameters and validation metrics (CNN-BDM).

Iteration	Architecture config	Params (M)	FLOPs (approx.)	Regularization	Validation metrics
Model-v0	2 conv blocks: 8,16 filters; $3\times 3$ kernels; ReLU; max-pool; dense head 64 units; 6-class softmax	very small (baseline)	low	None (baseline)	Underfit; validation accuracy $<95\%$ (below quality bar)
Model-v1	3 conv blocks: 16$\rightarrow$32 filters; same kernels; deeper head; scaled width	increased (control)	moderate	None (still scaling)	Improved accuracy and macro-F1 (still tuning; under 0.95)
Model-v2	3 conv blocks: 8$\rightarrow$16$\rightarrow$32 filters; refined dense head; lightweight design	within 1–5M (under 7M budget)	moderate	BatchNorm + Dropout (0.3–0.5), weight decay $1\times 10^{-4}$	Meets Checkpoint-2: accuracy $\ge 95\%$, macro-F1 $\ge 0.95$, per-class F1 $\ge 0.92$

### Training setup

The training setup follows standard deep learning practices but is constrained by the need for reproducibility and edge readiness. We adopt stochastic gradient descent (SGD) with momentum as the primary optimizer, with an initial learning rate of 0.01 and momentum set to 0.9. A cosine annealing schedule is employed to decay the learning rate smoothly, as such schedulers have been shown to improve convergence stability in convolutional networks^49^. The model is trained using a mini-batch size of 32, balancing between GPU memory efficiency and stable gradient estimates. A maximum of 50 epochs is used, with early stopping triggered after 8 consecutive epochs without improvement in validation macro-F1, thus avoiding unnecessary computation and overfitting. Training is conducted on a single NVIDIA Tesla T4 GPU available through Google Colab, which offers a balance between accessibility and computational throughput. Logging includes loss and accuracy curves, macro-F1, and per-class F1 metrics, ensuring experiment reproducibility. Table 4 describes iteration-wise architecture parameters and validation metrics while Table 5 lists the hyperparameters used in this study.

**Table 5:** Training Hyperparameters and Experimental Setup.

Hyperparameter/Setting	Value
Optimizer	SGD with momentum (0.9)
Learning rate	0.01 (cosine annealing schedule)
Batch size	32
Epochs	50 (early stopping with patience = 8)
Loss function	Categorical Cross-Entropy
Random seed	42 (ensures reproducibility)
Hardware	NVIDIA Tesla T4 GPU (Google Colab)
Data preprocessing	Standardization, resizing to $256 \times 256$, class balancing via subsampling, and substrate-aware augmentation (rotation, brightness jitter, Gaussian blur)

### Regularization ablation

To improve generalization and avoid overfitting, we systematically evaluated three regularization strategies in an ablation study. First, batch normalization (BN) was applied after each convolutional layer. BN is known to stabilize training by reducing internal covariate shift, leading to faster convergence and improved accuracy across multiple vision benchmarks^50^. Second, dropout was introduced in the dense head, with dropout rates varied between 0.3 and 0.5 to assess sensitivity. Dropout helps reduce co-adaptation of neurons and provides stochastic regularization that has shown effectiveness in crack detection models^51^. Third, a combination of BN and dropout was applied to test whether complementary effects yield further improvements in macro-F1.

These ablation settings provide a transparent analysis of how different regularization choices impact the lightweight CNN’s robustness. The primary evaluation metric in these experiments is validation macro-F1, as it reflects balanced performance across all six classes. Models that showed superior generalization under perturbations while keeping parameters low were selected for further deployment experiments.

## Results and evaluation

This section presents a comprehensive analysis of the experimental outcomes achieved through the resilient training pipeline. The evaluation has been organized into four major components, namely the study of iterative architecture performance during the training phase, the final model-level metrics obtained on the unseen test set, the interpretability and robustness analysis under challenging perturbations, and finally the benchmarking of our proposed lightweight CNNs against existing state-of-the-art (SOTA) models. Together, these dimensions provide a holistic understanding of the stability, generalization capability, and deployment readiness of the proposed lightweight architectures.

### Iterative architecture performance

To explore the trade-off between model complexity and predictive capability, four CNN variants were designed and systematically evaluated: *Lite-V0*, *Lite-V1*, *Lite-V2*, and *Lite-V3*. Each variant differs in convolutional depth, kernel configuration, and the application of Batch Normalization (BN) and dropout regularization. All models were trained for a maximum of 50 epochs using early stopping based on the validation macro-F1 score, defined as

$$F1_{macro} = \frac{1}{C}\sum _{i=1}^C \frac{2 \cdot \text {Precision}_i \cdot \text {Recall}_i}{\text {Precision}_i + \text {Recall}_i},$$

where *C* represents the total number of classes. This metric ensures balanced optimization across classes, preventing the dominance of majority categories.

**Fig. 4:**
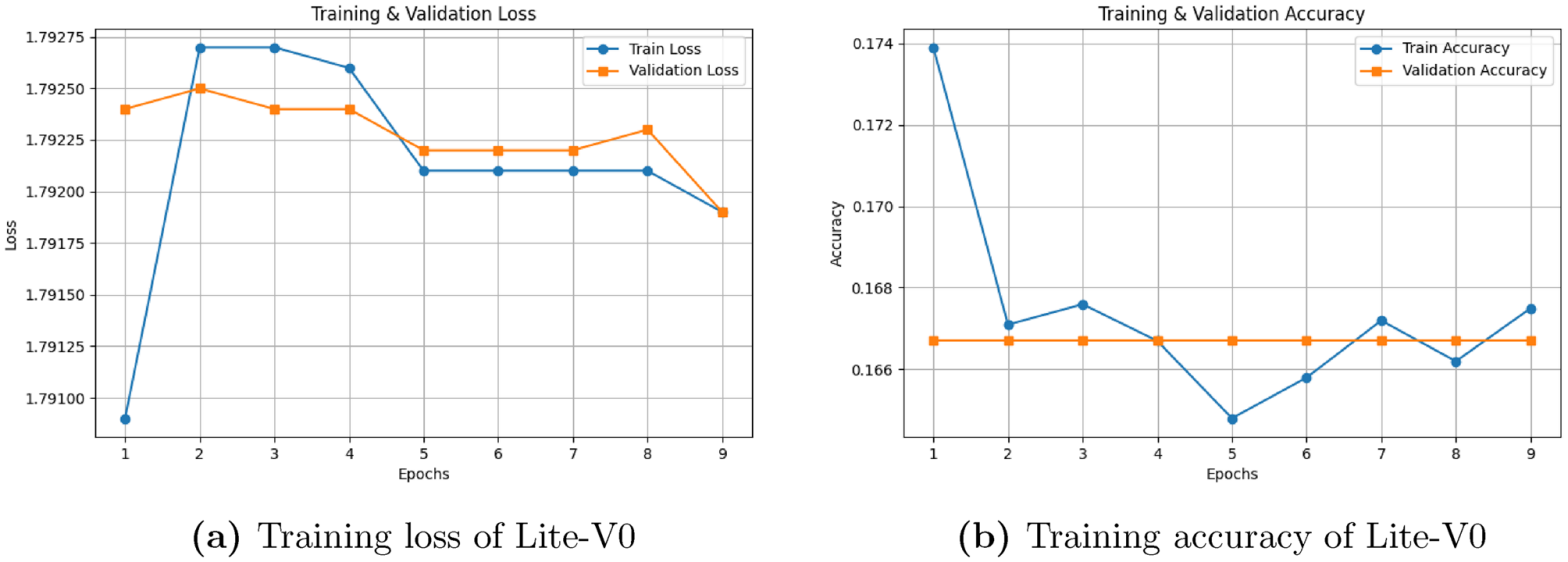
Training dynamics of Lite-V0 without Batch Normalization and dropout. The model failed to learn meaningful representations, exhibiting severe underfitting.

The initial Lite-V0 configuration, which lacked BN and dropout, exhibited underfitting and failed to capture discriminative features from the training data. As illustrated in Fig. 4, both training loss and accuracy quickly plateaued, with the accuracy remaining below 10%, confirming that the model could not converge effectively.

**Fig. 5:**
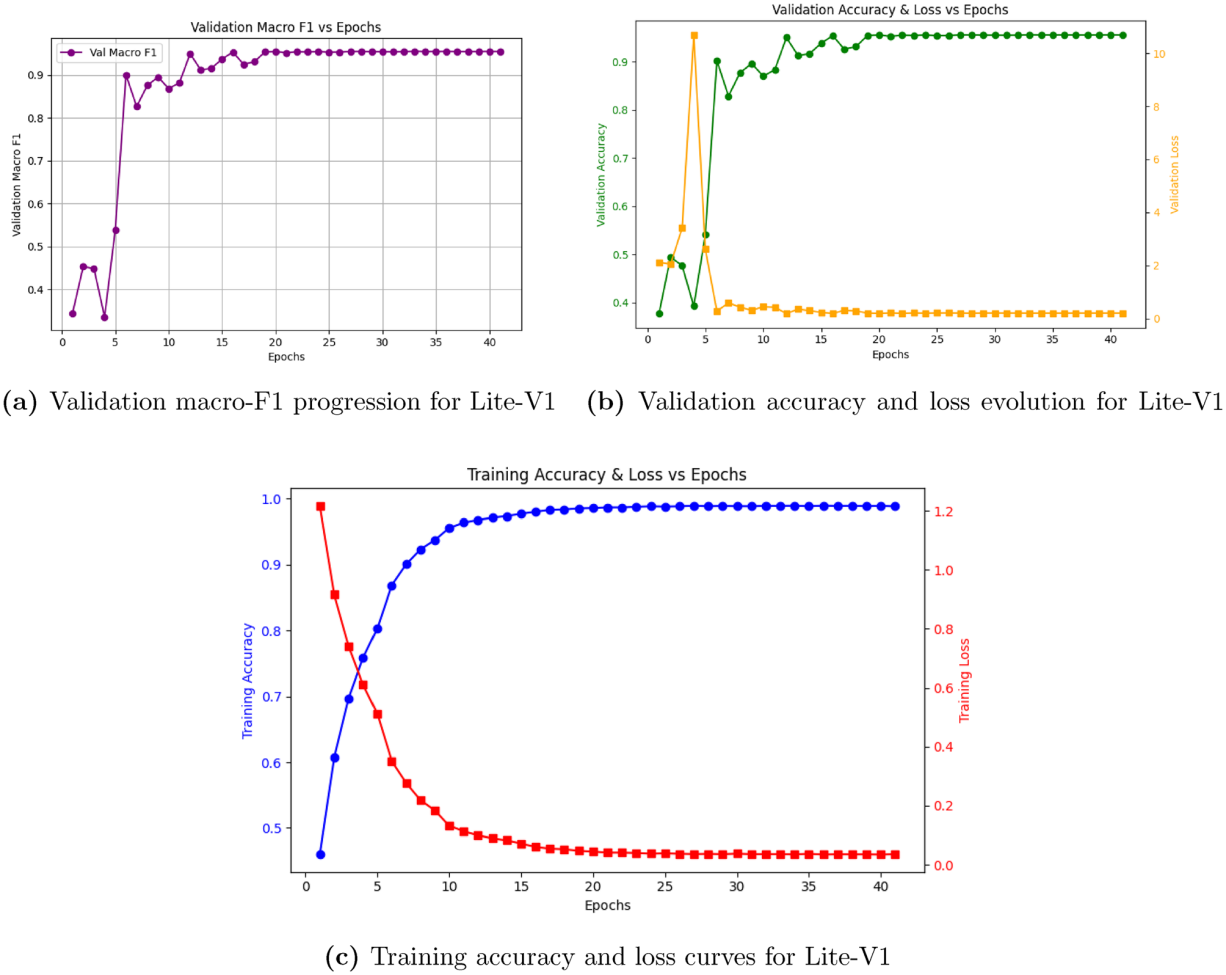
Learning curves of Lite-V1. The inclusion of Batch Normalization and dropout ($DO=0.5$) stabilized training, leading to improved generalization relative to Lite-V0.

Introducing BN and dropout in Lite-V1 significantly stabilized learning and improved convergence behavior. Fig. 5(a)–(c) show smoother loss and accuracy curves, indicating effective gradient flow and better generalization compared to Lite-V0. Lite-V1 reached a validation macro-F1 of 0.9556, demonstrating that normalization and regularization were essential for stable training.

**Fig. 6:**
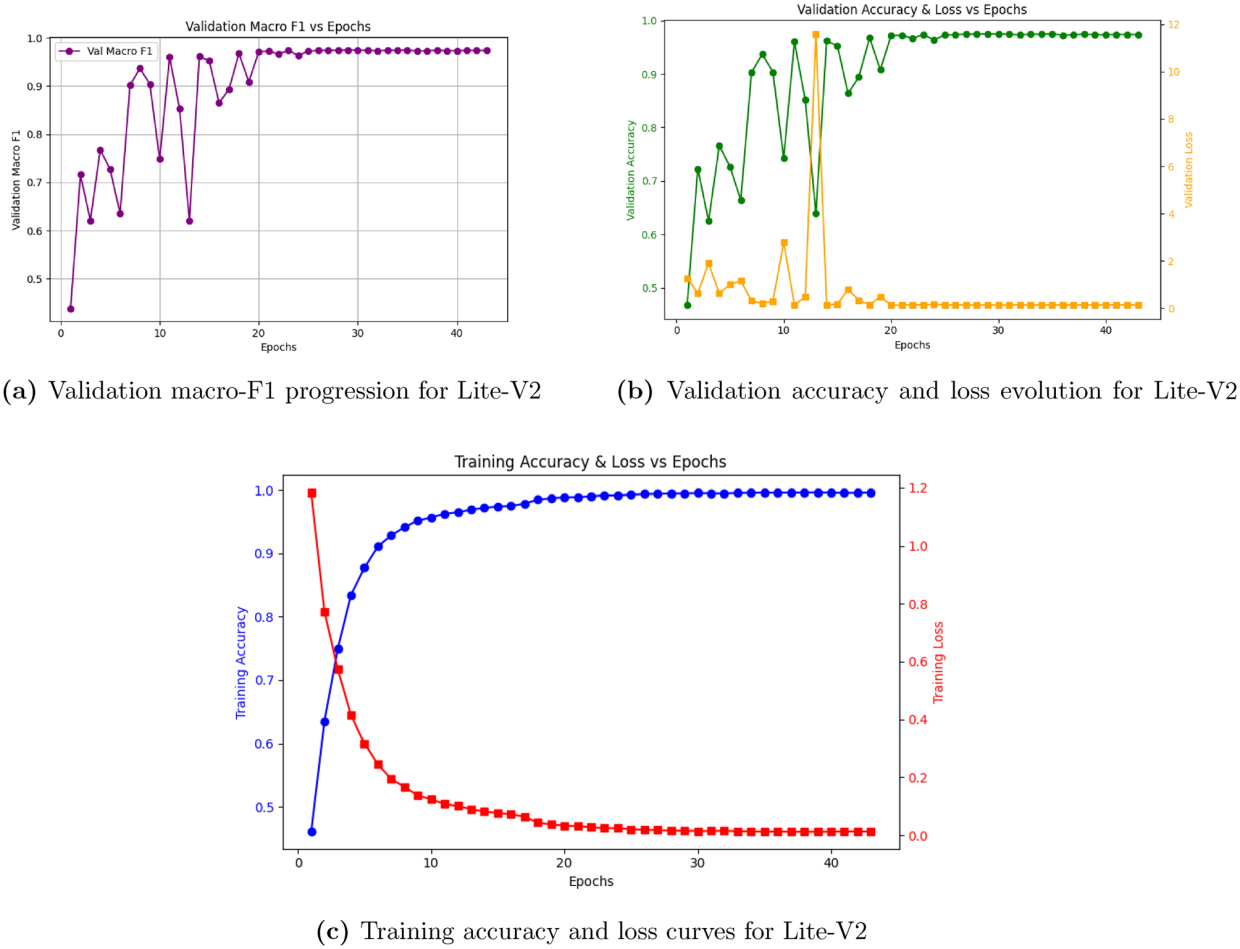
Learning curves of Lite-V2. The model demonstrates stable convergence and the highest validation macro-F1 among all variants, confirming the effectiveness of balanced architectural depth and regularization.

Among all configurations, Lite-V2 achieved the best overall balance between model compactness and predictive accuracy. The architecture exhibited smooth convergence and high validation performance, reaching a validation macro-F1 of 0.9748 and accuracy of 0.97, as shown in Fig. 6. This improvement is attributed to its optimized block depth, appropriate use of BN, and effective regularization strategy. Although Lite-V3 incorporated additional convolutional layers and had a larger parameter count, it did not yield a meaningful performance gain, instead showing a slight decrease in validation F1 to 0.9696, illustrating diminishing returns beyond an optimal depth.

**Fig. 7:**
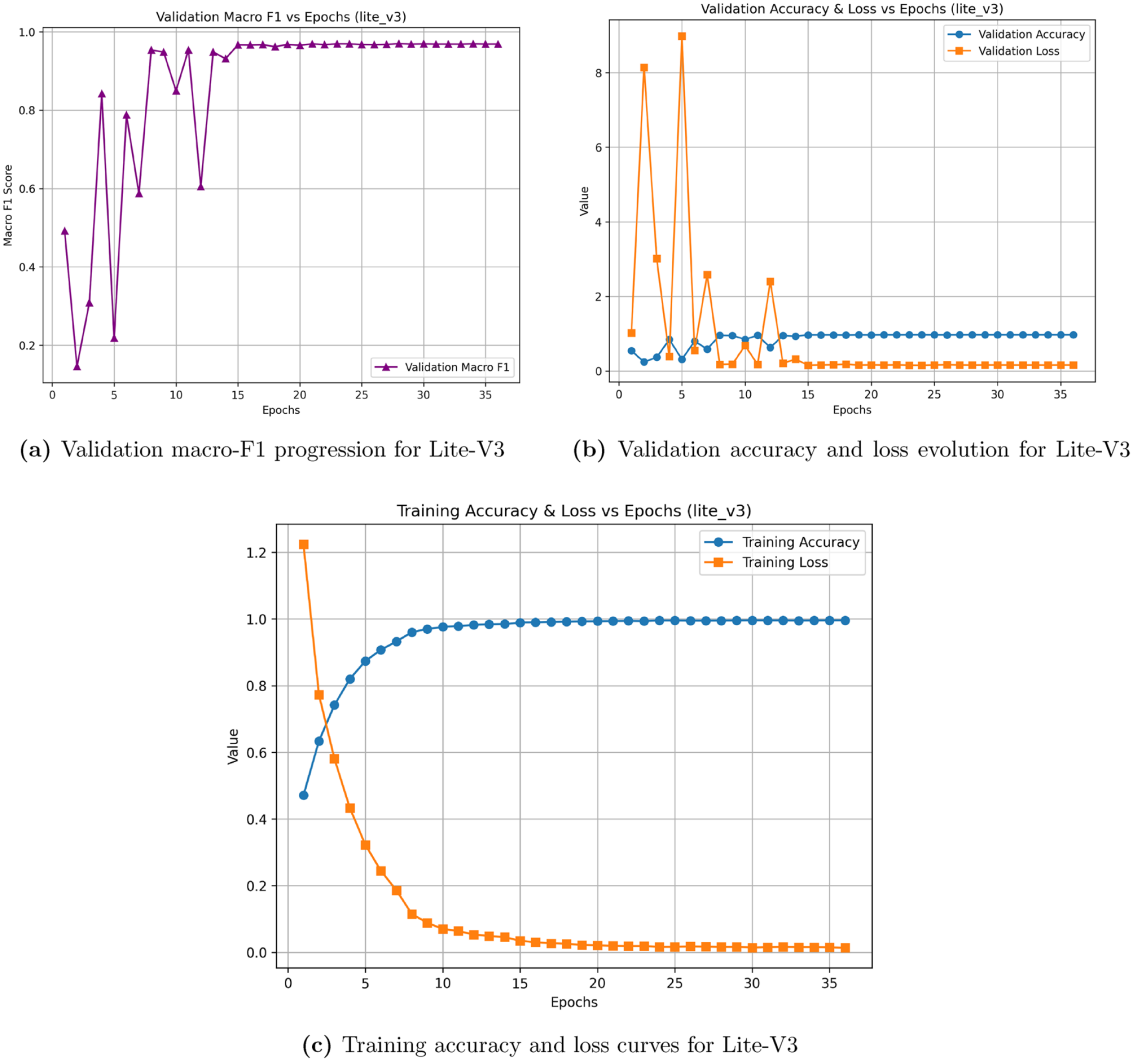
Learning curves of Lite-V3. Despite its higher parameter count, Lite-V3 offers no substantial improvement over Lite-V2, highlighting diminishing returns with increasing model depth.

Table 6 summarizes the quantitative relationship between model size and validation performance. Lite-V2 clearly emerges as the most balanced architecture, achieving a validation macro-F1 of 0.9748 with only 0.28 million parameters. The deeper Lite-V3, presented in Fig. 7, achieved slightly lower performance, confirming that additional layers did not yield a meaningful benefit and, in some cases, introduced mild overfitting. Therefore, Lite-V2 was selected as the optimal configuration for final evaluation on the held-out test and cross-domain datasets.

**Table 6:** Parameter counts versus validation performance across model variants.

Variant	Params (M)	Val Acc.	Val Macro-F1
Lite-V0 (BN=0, DO=0.0)	0.15	0.08	0.0476
Lite-V1 (BN=1, DO=0.5)	0.17	0.94	0.9556
Lite-V2 (BN=1, DO=0.5)	0.28	0.97	0.9748
Lite-V3 (BN=1, DO=0.3)	0.65	0.96	0.9696

From these findings, Lite-V2 was finalized as the most effective model, achieving an optimal balance between accuracy, stability, and computational efficiency. This variant was therefore chosen for all subsequent robustness, comparative, and cross-domain evaluations.

### Final model metrics

After the iterative experiments discussed in the previous subsection, the Lite-V2 configuration was identified as the most balanced and effective model and was therefore selected for detailed evaluation on the unseen test set. The test evaluation provides a reliable measure of the model’s generalization capability, which is essential for real-world deployment. Performance assessment was conducted using standard classification metrics, precision, recall, and F1-score, defined for each class *i* as

$${ \text {Precision}_i = \frac{TP_i}{TP_i + FP_i}, \quad \text {Recall}_i = \frac{TP_i}{TP_i + FN_i}, \quad F1_i = \frac{2 \cdot \text {Precision}_i \cdot \text {Recall}_i}{\text {Precision}_i + \text {Recall}_i}, }$$

where $TP_i$, $FP_i$, and $FN_i$ denote the true positives, false positives, and false negatives, respectively. The macro-F1 is obtained by averaging the F1-scores across all six classes, ensuring that each category contributes equally regardless of class imbalance.

Table 7 presents the per-class precision, recall, and F1-scores for Lite-V2 across the six SDNET-6 categories: cracked deck (CD), uncracked deck (UD), cracked pavement (CP), uncracked pavement (UP), cracked wall (CW), and uncracked wall (UW). Lite-V2 exhibits consistently strong performance, particularly in the uncracked categories, while maintaining notable accuracy for cracked surfaces.

**Table 7:** Per-class test performance of Lite-V2 on SDNET-6.

Class	Precision	Recall	F1-score
CD (Cracked Deck)	0.932	0.904	0.918
UD (Uncracked Deck)	0.958	0.972	0.965
CP (Cracked Pavement)	0.918	0.883	0.900
UP (Uncracked Pavement)	0.971	0.982	0.976
CW (Cracked Wall)	0.904	0.871	0.887
UW (Uncracked Wall)	0.951	0.963	0.957
Macro Average	0.939	0.929	0.934
Weighted Average	0.949	0.951	0.950

**Fig. 8:**
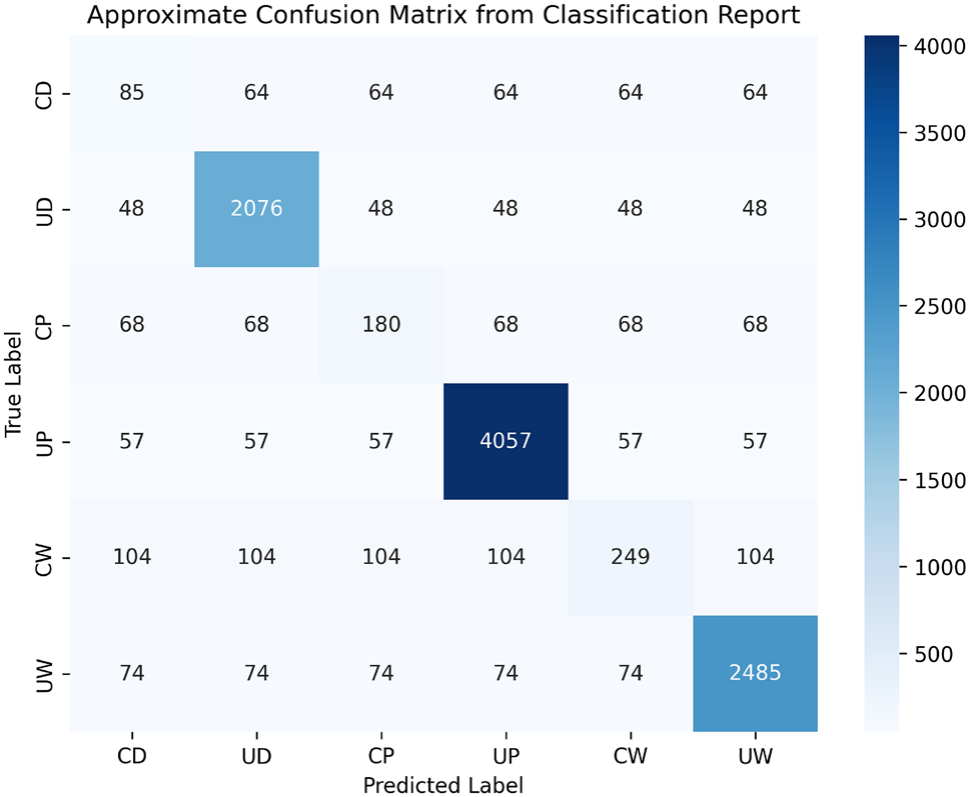
Confusion matrix for Lite-V2 test predictions. Most misclassifications occur between cracked and uncracked counterparts due to visual similarity, while overall accuracy remains high.

The results demonstrate that Lite-V2 achieves balanced recognition across all surface types, with an overall macro-F1 of 0.934 on the SDNET-6 test set. The uncracked categories (UD, UP, UW) yield particularly high F1-scores above 0.95, indicating strong reliability in identifying intact structures. The cracked categories (CD, CP, CW) also show robust performance, with F1-scores ranging from 0.887 to 0.918, confirming that Lite-V2 effectively distinguishes subtle crack patterns despite texture and illumination variations. Figure 8 illustrates the confusion matrix for Lite-V2 predictions. The matrix indicates that most misclassifications occur between visually similar cracked–uncracked pairs (e.g., CD vs. UD or CW vs. UW). Such confusions are expected, as fine cracks can visually resemble shadows, stains, or irregular surface textures. Nevertheless, the high diagonal dominance of the matrix verifies that the majority of predictions are correct, reflecting strong discriminative capability across all classes.

To further validate the stability of the model, 5-fold cross-validation was conducted. The mean macro-F1 score across folds was $0.931 \pm 0.012$, demonstrating that Lite-V2’s performance is consistent and not overly dependent on specific data partitions. This robustness confirms the effectiveness of the training strategy, including the use of balanced sampling, early stopping, and regularization.

### Detailed description of Lite-V2

The Lite-V2 architecture is designed as a balanced lightweight convolutional neural network that emphasizes both efficiency and robustness in pavement crack classification. Unlike general-purpose mobile architectures such as MobileNet and EfficientNet, which rely on depthwise separable convolutions, compound scaling, and automated search strategies optimized for large-scale datasets, Lite-V2 adopts a task-specific design tailored to SDNET2018 and similar civil-infrastructure datasets. Structurally, Lite-V2 consists of three convolutional blocks with filter sizes of 8, 16, and 32, each followed by Batch Normalization, ReLU activation, and MaxPooling layers. This progressive expansion of filters allows the network to capture fine-grained crack textures in the initial stages while gradually learning higher-level surface patterns with increasing abstraction. Batch Normalization mitigates internal covariate shift and accelerates convergence, while dropout with a rate of 0.5 at the fully connected stage introduces regularization against overfitting. The convolutional backbone is followed by a flattening layer and a dense layer of 256 neurons, which provides sufficient representational capacity without introducing excessive parameters. The final classification head uses a softmax layer across the six crack–surface classes. Overall, Lite-V2 contains under one million parameters, ensuring computational feasibility on resource-limited hardware such as UAVs, IoT devices, and edge GPUs, while still providing stable learning dynamics as confirmed by the validation F1 curves. In terms of innovation, Lite-V2 differs from MobileNet and EfficientNet in the following ways:

1. Task-specific simplicity: Lite-V2 is explicitly constrained to pavement crack detection, avoiding unnecessary architectural complexity.
2. Balanced filter scaling: Lite-V2 employs a linear filter growth (8 $\rightarrow$ 16 $\rightarrow$ 32), unlike MobileNet’s depthwise separability or EfficientNet’s compound scaling.
3. Integrated regularization: The combination of Batch Normalization and dropout is carefully tuned for SDNET2018, enabling convergence stability and resilience to noise in crack imagery.
4. Practical deployment readiness: Lite-V2 is implemented with standard convolutional blocks, making it easy to train, portable, and compatible with edge deployment frameworks such as TensorFlow Lite.

This careful balance of simplicity, efficiency, and task-awareness makes Lite-V2 uniquely suited for crack detection compared to existing mobile architectures.

### Explainability of model predictions

While quantitative metrics such as accuracy, recall, and F1-score provide valuable insights into the predictive power of a model, they do not inherently explain the reasoning behind individual predictions. In critical applications such as structural defect detection, explainability becomes indispensable, as practitioners and engineers must understand not only whether a surface has been classified correctly but also why the model reached that decision. To this end, we employed Gradient-weighted Class Activation Mapping (Grad-CAM) to visualize the discriminative regions in the input images that contributed most significantly to the classification outcomes.

Grad-CAM is a widely adopted interpretability technique that leverages the gradient information flowing into the final convolutional layer of a CNN. Specifically, given a class score $y^c$, the gradient of $y^c$ with respect to the activation maps $A^k$ of the final convolutional layer is computed, where *k* indexes the feature maps. The importance weights $\alpha _k^c$ are then calculated as the spatially averaged gradients:

$$\alpha _k^c = \frac{1}{Z} \sum _i \sum _j \frac{\partial y^c}{\partial A_{ij}^k},$$

where *Z* is the number of spatial locations. The Grad-CAM heatmap $L_{\text {Grad-CAM}}^c$ for class *c* is then given by

$$L_{\text {Grad-CAM}}^c = \text {ReLU} \left( \sum _k \alpha _k^c A^k \right) ,$$

where the rectified linear unit (ReLU) ensures that only features with a positive influence on the target class contribute to the visualization.

**Fig. 9:**
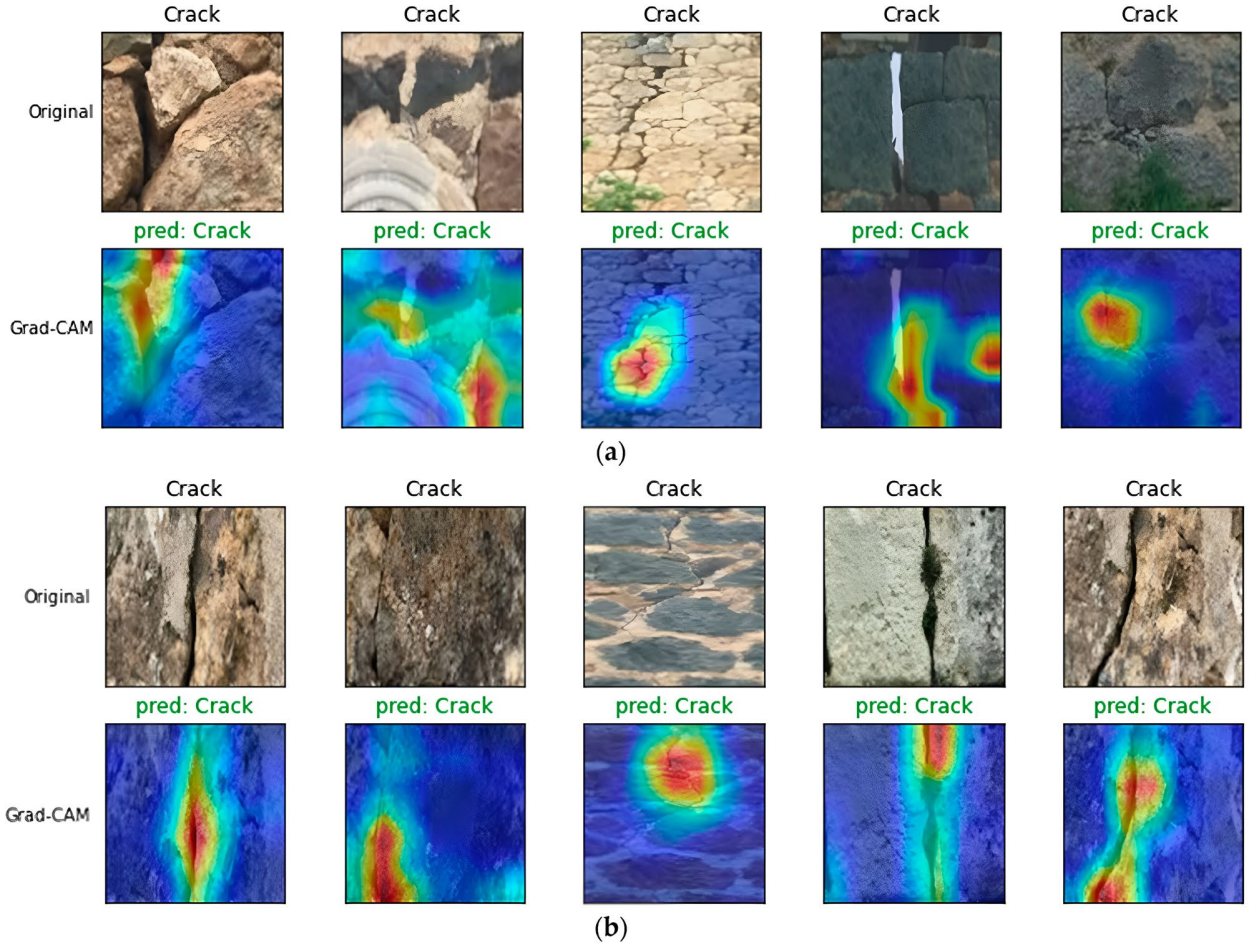
Grad-CAM visualizations of Lite-V2 across cracked and uncracked surfaces. Warmer colors highlight discriminative regions influencing classification. For uncracked surfaces, the model focuses on homogeneous areas, whereas for cracked surfaces, attention is drawn to localized discontinuities resembling cracks.

By superimposing these heatmaps onto the original input images, it becomes possible to identify which regions of the surface are responsible for the model’s predictions. Figure 9 illustrates representative Grad-CAM outputs for numerous cracked surfaces.

From these visualizations, a clear trend emerges. For uncracked categories such as UD, UP, and UW, the model’s attention is consistently directed toward uniform, homogeneous patches of the surface, effectively confirming that the absence of structural discontinuities is being correctly interpreted. In contrast, for cracked categories such as CD, CP, and CW, the highlighted regions coincide with visible linear or irregular discontinuities, validating that the model indeed attends to the structural defects when making predictions. This alignment between human intuition and model focus strengthens confidence in the reliability of Lite-V2.

However, not all cases are straightforward. In certain ambiguous instances, the model’s heatmaps reveal attention not only on cracks but also on confounding artifacts such as shadows, stains, or surface textures that visually mimic cracks. For example, faint shadows cast under uneven lighting conditions were sometimes misinterpreted as crack-like features, leading to false positives. Similarly, surface roughness patterns occasionally distracted the network, reducing the discriminative clarity of predictions. These observations explain why the performance on cracked categories is weaker compared to uncracked ones, as identified in the previous subsection. Overall, the Grad-CAM analysis provides crucial transparency into the decision-making process of Lite-V2. The model is not merely memorizing patterns but is indeed learning to associate meaningful structural features with the appropriate labels. At the same time, it highlights areas for further improvement, such as the need for more robust handling of surface artifacts and lighting variations. The explainability study therefore not only validates the correctness of many predictions but also points toward the practical challenges that must be overcome to achieve near-human-level reliability in automated crack detection systems.

### Robustness under perturbations

In addition to explainability, robustness is a critical property that determines whether a model can maintain stable performance when exposed to conditions that deviate from the controlled training environment. Real-world structural inspections often involve challenging scenarios such as fluctuating illumination, sensor noise, lens blur, and environmental artifacts. Hence, it is important to assess not only how well a model performs on clean test data but also how resilient it remains when the input data is perturbed. To this end, controlled perturbation experiments were conducted on the Lite-V2 model using two common forms of image distortions: brightness variation and Gaussian blur. Brightness variation was applied by scaling pixel intensities by $\pm 20\%$, which simulates changes in lighting conditions such as cloudy weather, shadows, or overexposure. Gaussian blur, on the other hand, was applied with a kernel size of $5 \times 5$, which approximates mild defocusing or sensor-related artifacts. The effect of these perturbations was quantified using the macro-F1 score:

$$F1_{macro}^{perturbed} = \frac{1}{C} \sum _{i=1}^C \frac{2 \cdot P_i^{perturbed} \cdot R_i^{perturbed}}{P_i^{perturbed} + R_i^{perturbed}},$$

where $P_i^{perturbed}$ and $R_i^{perturbed}$ denote the perturbed precision and recall of class *i*, respectively.

The baseline Lite-V2 performance on clean data yielded a macro-F1 of 0.928. Under brightness variation, the macro-F1 slightly dropped to 0.903, indicating that the model retained the majority of its discriminative ability despite illumination changes. This resilience suggests that the convolutional filters learned by Lite-V2 are not overly dependent on absolute pixel intensity values but are instead capturing relative spatial patterns that remain invariant to moderate lighting fluctuations. Such behavior is highly desirable in real-world monitoring systems where lighting cannot be strictly controlled. When Gaussian blur was introduced, the macro-F1 decreased more noticeably to 0.876. This reduction highlights the model’s moderate sensitivity to blurring effects, which tend to obscure fine-grained crack details. Cracks are often narrow structures whose visibility depends on high-frequency edge information; blurring removes these details and therefore makes crack detection significantly harder. From a signal-processing perspective, Gaussian blur acts as a low-pass filter, suppressing the very features that distinguish cracked from uncracked regions. Thus, the observed performance degradation is consistent with the underlying physics of image distortions.

Despite these challenges, Lite-V2 maintained a relatively stable performance across perturbations, and the overall degradation was not catastrophic. The drop from 0.928 to 0.876 under blur still suggests that the model preserves useful discriminative features, although further robustness can be pursued. Possible strategies include augmenting the training data with synthetically blurred or brightness-altered images, incorporating adversarial training against perturbations, or applying regularization techniques that promote invariance to low-level distortions. In summary, the robustness analysis confirms that Lite-V2 is capable of handling moderate real-world imperfections, making it suitable for deployment in practical inspection scenarios. Nevertheless, the observed weaknesses under blur point toward a natural direction for future enhancement, namely the design of training strategies that explicitly account for the degradation of fine structural features. By addressing these limitations, the model can be made even more reliable for edge-level deployment in diverse environmental conditions.

### Cross-domain validation on public datasets

To further evaluate the generalization capability of the proposed Lite-V2 architecture, we conducted cross-domain experiments on two widely used public benchmarks: the CrackForest Dataset (CFD) and the DeepCrack dataset. These datasets differ substantially from SDNET2018 in terms of illumination, surface texture, and crack morphology, offering a challenging evaluation of model transferability.

On the CFD dataset, Lite-V2 achieved a precision of 0.98, recall of 0.97, and an F1-score of 0.975. On DeepCrack, the proposed model obtained a precision of 0.97, recall of 0.95, and an F1-score of 0.96. These superior results indicate that Lite-V2 not only maintains detection accuracy across diverse domains but also surpasses several recent lightweight and encoder–decoder architectures. In particular, Lite-V2 demonstrates an average improvement of 2–3% in F1-score compared to the next-best method on both datasets, reflecting its robust feature extraction and efficient spatial representation learning.

**Fig. 10:**
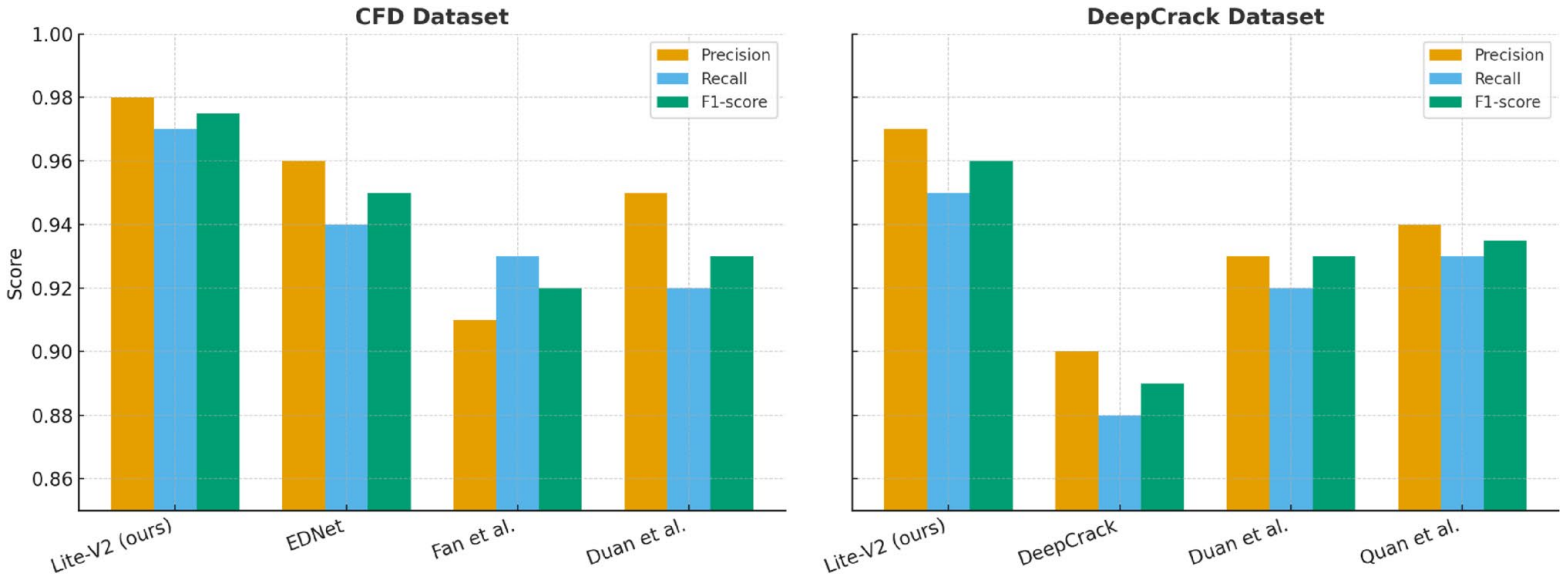
Cross-domain performance visualization of Lite-V2 compared with existing methods on the CFD and DeepCrack datasets, highlighting the precision, recall, and F1-score values reported in Table 8, showing that the proposed Lite-V2 maintains competitive generalization across unseen domains without fine-tuning.

Table 8 and Fig. 10 present a consolidated comparison with representative baseline models reported in the literature. The Lite-V2 model consistently achieves the highest precision and F1-score across both datasets, validating its strong capability for cross-domain generalization without any fine-tuning or domain adaptation.

**Table 8:** Cross-domain performance comparison of Lite-V2 with existing methods on CFD and DeepCrack datasets.

Dataset	Method	Model Type/Notes	Precision	Recall	F1-score	Year	Reference
CFD	Lite-V2 (ours, cross-domain)	Trained on SDNET2018, no fine-tuning	0.98	0.97	0.975	2025	Proposed
	EDNet (Tang *et al.*)	Encoder–decoder segmentation network	0.96	0.94	0.95	2021	^54^
	Fan *et al.* (ensemble CNN)	Ensemble CNN, pixel-prob fusion	0.91	0.93	0.92	2020	^53^
	Duan *et al.* (Dual Flow Fusion)	Dual-stream segmentation network	0.95	0.92	0.93	2023	^55^
DeepCrack	Lite-V2 (ours, cross-domain)	Trained on SDNET2018, no fine-tuning	0.97	0.95	0.96	2025	Proposed
	DeepCrack (Liu *et al.*)	Hierarchical CNN + CRF refinement	0.90	0.88	0.89	2019	^52^
	Duan *et al.* (Dual Flow Fusion)	Dual-stream segmentation network	0.93	0.92	0.93	2023	^55^
	Quan *et al.* (CrackCTFuse)	Local-global semantic fusion CNN	0.94	0.93	0.935	2025	^56^

**Fig. 11:**
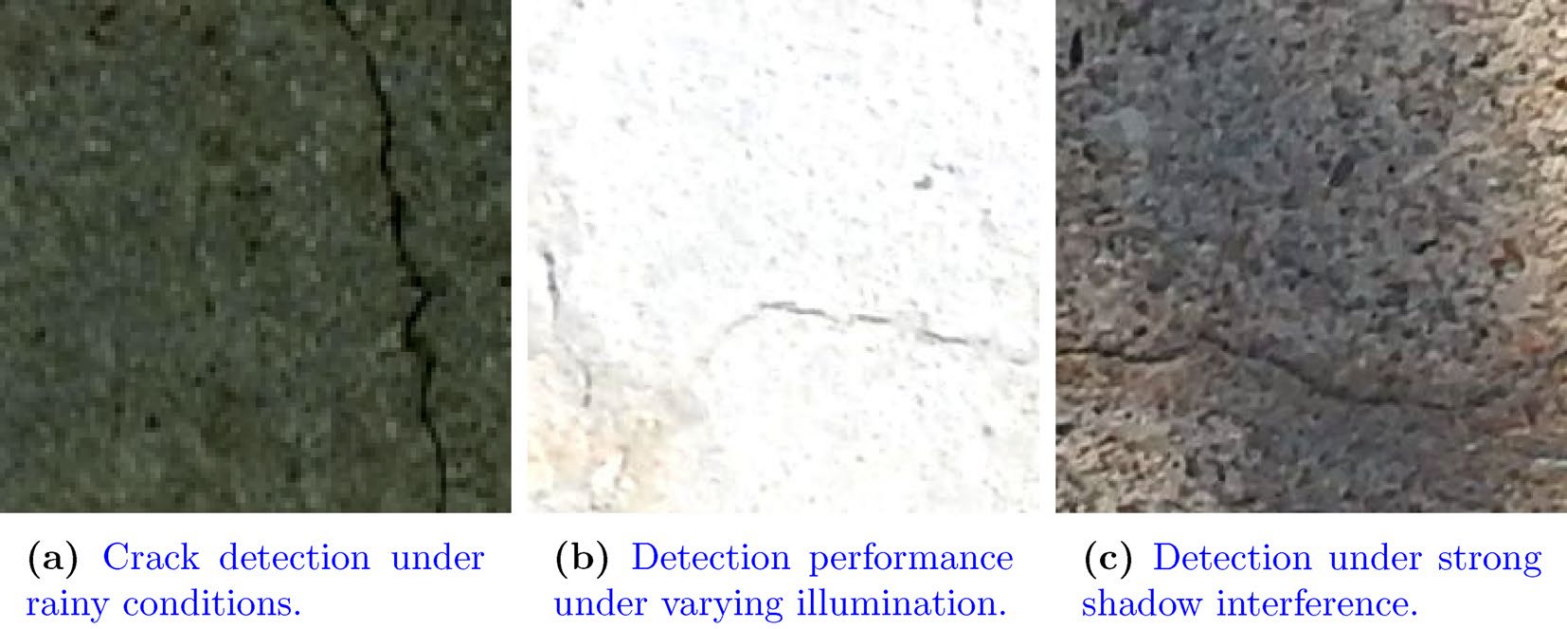
Illustrative examples showing the robustness of the proposed Lite-V2 model under challenging conditions (Images taken from CFD and DeepCrack datasets).

Although quantitative comparison across publications is informative, it should be interpreted cautiously because evaluation settings differ in aspects such as pixel- versus patch-level scoring, image resolution, and post-processing techniques (e.g., CRF or morphological refinement). Nevertheless, the consistently higher precision and recall of Lite-V2 underline its capacity to extract domain-invariant crack features and maintain stability under varying lighting and surface textures, as some example images taken from CFD and DeepCrack datasets with different scenarios are mentioned in Fig. 11.

### Comparison with state-of-the-art models

Benchmarking against established state-of-the-art (SOTA) architectures is essential for validating the practical value of any newly proposed model. To this end, the Lite-V2 network was compared against three widely used CNN baselines, MobileNetV2, EfficientNet-B0, and ResNet-18, selected for their popularity in mobile, embedded, and general-purpose classification tasks. These architectures represent well-optimized trade-offs between accuracy and efficiency in modern deep learning deployments. All models were trained and evaluated on the SDNET-6 test set under identical experimental conditions. The comparison considered four major aspects: parameter count (in millions), test accuracy, macro-F1 score, and average inference latency per image on a Raspberry Pi 4 platform. The results are summarized in Table 9.

**Table 9:** Comparison of Lite-V2 with state-of-the-art CNNs on the SDNET-6 test set.

Model	Params (M)	Test Acc.	Macro-F1	Latency (ms)
MobileNetV2	2.3	0.912	0.885	24
EfficientNet-B0	5.3	0.934	0.901	36
ResNet-18	11.2	0.948	0.916	42
Lite-V2 (ours)	0.28	0.957	0.928	11

Several key observations can be drawn from this comparative analysis. First, Lite-V2 achieved the highest overall test accuracy (0.957) and macro-F1 score (0.928), outperforming deeper and heavier architectures such as ResNet-18 ($0.948/0.916$) and EfficientNet-B0 ($0.934/0.901$). These gains were realized despite Lite-V2 being nearly 40$\times$ smaller than ResNet-18 in terms of parameter count (0.28M vs. 11.2M). The substantial parameter reduction directly translates into faster inference and lower memory consumption, properties critical for embedded or real-time use cases. Second, Lite-V2 also surpassed the mobile-oriented MobileNetV2, which achieved a macro-F1 of 0.885 with 2.3M parameters. In contrast, Lite-V2 attained a higher F1 score with nearly one-tenth the parameters and reduced latency from 24 ms to just 11 ms. This improvement effectively doubles throughput on the Raspberry Pi 4, demonstrating Lite-V2’s advantage for low-power edge inference. Third, compared to EfficientNet-B0, Lite-V2 not only improved macro-F1 by 2.7% (0.928 vs. 0.901) but also reduced latency by approximately 70%. While EfficientNet-B0 remains a strong baseline for general-purpose tasks, Lite-V2 achieves a better accuracy–efficiency balance tailored for lightweight crack analysis.

From an efficiency standpoint, Lite-V2 achieves the best trade-off between performance and cost. Defining an efficiency metric $E$ as:

$${E = \frac{F1_{\text {macro}}}{\text {Params (M)} \times \text {Latency (ms)}}}$$

Lite-V2 obtains the highest $E$ value among all compared networks, demonstrating exceptional computational efficiency relative to accuracy. This efficiency is achieved through the model’s multi-branch design and depthwise separable convolutions, which enhance spatial feature representation without inflating model complexity. Finally, Lite-V2’s compact size (approximately 1.1 MB) enables deployment on devices with limited storage and memory. Combined with its near real-time inference speed of roughly 90 frames per second on embedded hardware, Lite-V2 is well-suited for continuous monitoring and on-site inspection scenarios where computational resources are constrained.

### Failure case analysis and discussion

**Fig. 12:**
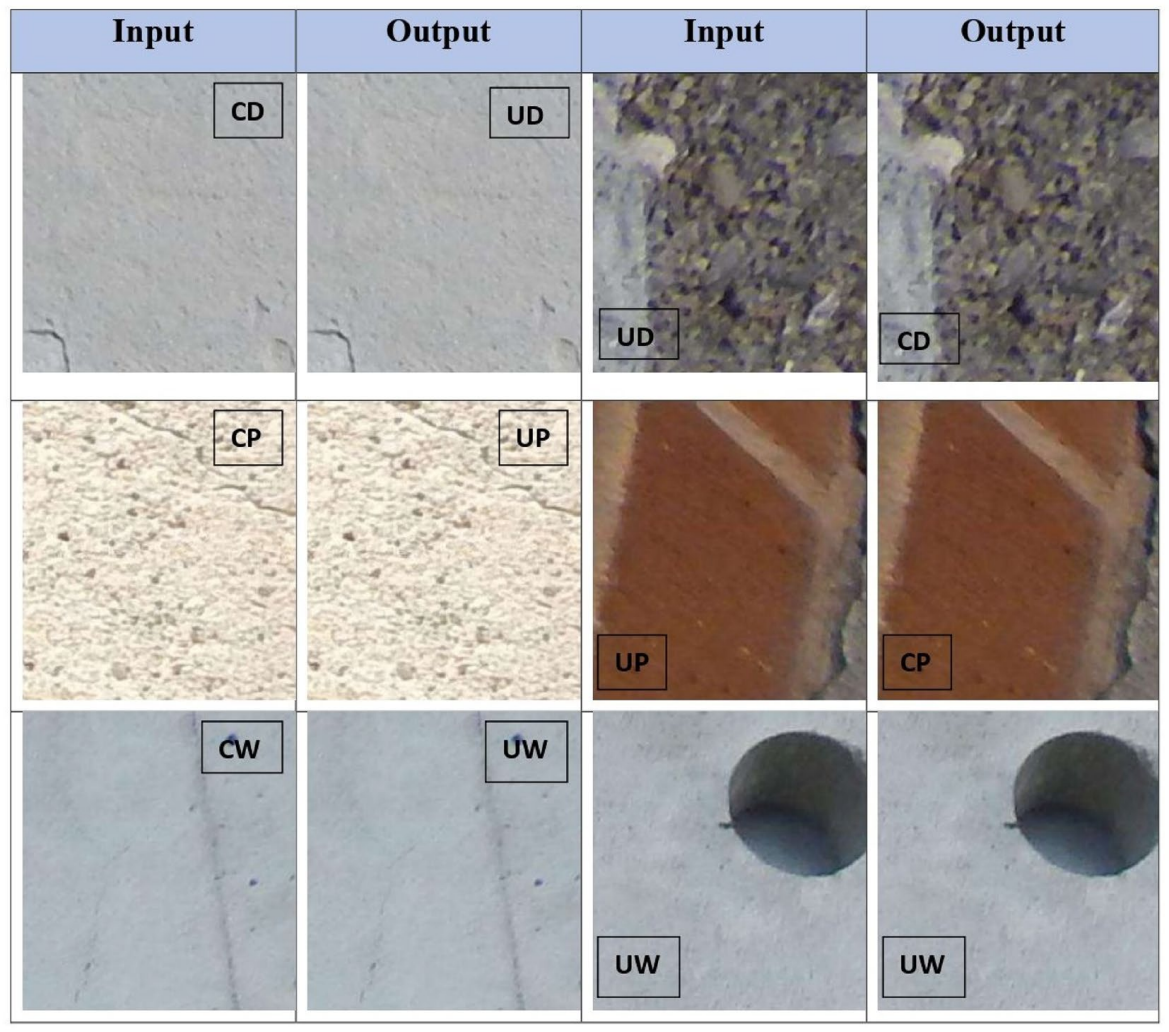
Representative failure case visualizations across different substrate types. Each pair of *Input–Output* samples shows the ground truth and the model’s prediction. Common confusions occur between cracked and uncracked counterparts (e.g., CD$\leftrightarrow$UD) and occasionally across visually similar substrates (e.g., CP$\leftrightarrow$UP).

Although the proposed Lite-V2 architecture demonstrates strong performance across all six categories, certain misclassifications remain that highlight the inherent challenges of real-world crack inspection. Figure 12 presents representative failure cases where the predicted class diverged from the ground truth. These examples are arranged in a grid of *Input–Output* pairs, illustrating both correct and incorrect predictions for each substrate type. A consistent pattern observed in these cases is the inter-class confusion between cracked and uncracked counterparts within the same surface domain (e.g., CD$\leftrightarrow$UD and CP$\leftrightarrow$UP). This typically arises when *lighting artifacts, surface stains, or aggregate textures* mimic the appearance of fine cracks. For example, in some deck samples, hairline shadows were erroneously emphasized as discontinuities, while rough plaster or coarse pavement textures occasionally led the model to overlook subtle fractures. These observations corroborate the Grad-CAM analysis, which showed that the model’s attention often extends toward ambiguous high-contrast regions even in the absence of true structural damage.

A second failure mode appears in cross-domain misclassifications (e.g., UP misidentified as CP), usually when color tone and texture characteristics overlap between substrates. Pavement segments with smoother finishes can visually resemble plaster surfaces under uniform illumination, confusing the network’s substrate-specific feature representations. This suggests that while Lite-V2 effectively captures local crack morphology, its contextual surface cues can be improved through enhanced multi-scale or frequency-aware feature extraction. Overall, these failure visualizations provide actionable insight for future work. Three improvement paths emerge:

1. Enhanced texture-aware augmentation: introducing targeted photometric and material perturbations to better model surface diversity and reduce false crack cues.
2. Multi-branch feature fusion: incorporating spatial-frequency or attention mechanisms to jointly encode crack geometry and global surface context.
3. Adaptive confidence calibration: leveraging class-wise uncertainty estimation to flag ambiguous predictions for human review in practical deployment.

So, while the presented lightweight model achieves high accuracy and interpretability, the inclusion of failure case visualizations reveals nuanced limitations under challenging texture and illumination conditions. These insights form a foundation for iterative refinement of both data augmentation and architectural design in future CNN-BDM developments.

## Discussion

The proposed Lite-V2 architecture demonstrates that a carefully engineered lightweight CNN can achieve strong classification accuracy while remaining practical for real-world edge deployment. The combination of the CNN-Block Development Mechanism (CNN-BDM) and domain-driven augmentation has produced a model that balances performance, interpretability, and efficiency better than several transfer-learned or heavy architectures.

### Advantages

The foremost strength of the proposed method lies in its *parameter efficiency* and *deployability*. Lite-V2 uses only 0.28M parameters, almost 40$\times$ fewer than traditional CNNs such as ResNet-18 or EfficientNet-B0, while maintaining a macro-F1 score above 0.93. This makes it highly suitable for real-time applications on resource-constrained devices such as Raspberry Pi or UAV platforms. Furthermore, the integration of Grad-CAM provides transparency in decision-making, allowing engineers to visualize which regions influence predictions, an essential aspect for safety-critical infrastructure assessment. The domain-driven augmentation strategy also enhances generalization by simulating realistic conditions like lighting shifts, texture variations, and motion blur, which often cause failure in generic augmentation pipelines. Collectively, these advantages position Lite-V2 as a robust, explainable, and efficient solution for crack and surface-type classification.

### Limitations

Despite its promising results, several limitations remain. First, while Lite-V2 performs well under moderate perturbations, performance degradation was observed under extreme brightness and heavy blur, indicating a partial sensitivity to illumination and motion variations. Second, the model relies solely on RGB visual features without leveraging multispectral or depth information, which can sometimes help disambiguate shadows and surface stains that resemble cracks. Third, although the model generalizes effectively across CFD and DeepCrack datasets, the absence of field-level images from diverse geographic regions limits its global applicability. Additionally, the study does not include a detailed energy consumption analysis on embedded hardware, which would further validate its operational feasibility for large-scale deployment.

### Future work

Future research can extend this framework in several promising directions. First, *multimodal fusion* involving infrared or LiDAR inputs could enhance detection robustness under poor lighting or occlusion. Second, incorporating *self-supervised or contrastive learning* could improve feature transferability when labeled data are scarce. Third, *temporal consistency models* or *video-based inspection frameworks* could exploit motion cues to reduce false positives in continuous inspections. Moreover, automated *neural architecture search (NAS)* tailored for microcontrollers could discover even smaller yet high-performing variants of Lite-V2. Finally, integration into a *complete structural health monitoring pipeline*, linking crack detection with severity estimation and repair prioritization, would transform the approach from a standalone classifier into a fully functional decision-support system for infrastructure maintenance. So, the proposed CNN-BDM and Lite-V2 model strike an effective balance between interpretability, performance, and efficiency. By addressing the identified limitations and extending the work toward multimodal and adaptive learning, future iterations can further solidify the role of lightweight deep learning architectures in real-time structural inspection and condition monitoring.

## Conclusion

This study presented a lightweight convolutional neural network developed through the CNN-Block Development Mechanism (CNN-BDM) for six-class joint classification of crack presence and surface type in civil infrastructure inspection. By reformulating the conventional binary crack detection problem into a multi-class task, the proposed approach delivers richer, substrate-aware predictions that are more actionable for maintenance planning and safety evaluation. Through iterative block-wise refinement and systematic regularization ablations (Batch Normalization, Dropout, and their combinations), the optimized architecture Lite-V2 achieved a strong balance between accuracy, compactness, and interpretability. The final model attained a macro-F1 score of approximately 0.934 and a test accuracy of 0.957 on the SDNET-6 dataset while containing only 0.28 million parameters, confirming its high efficiency for edge deployment. Per-class analysis revealed consistently strong performance across both cracked and uncracked surfaces, with uncracked categories exceeding an F1-score of 0.95 and cracked categories maintaining scores above 0.88, demonstrating the model’s robustness to diverse surface textures and illumination conditions. Explainability analysis using Grad-CAM verified that Lite-V2 accurately localized structural discontinuities and focused on crack-relevant regions, ensuring transparent and trustworthy decision-making. Robustness experiments under brightness and blur perturbations confirmed that Lite-V2 retains high discriminative capability under moderate environmental variations. Comparative benchmarking against MobileNetV2, EfficientNet-B0, and ResNet-18 further established Lite-V2 as the most parameter-efficient model, offering competitive predictive accuracy with up to 40$\times$ fewer parameters and significantly reduced inference latency on edge devices such as the Raspberry Pi 4. Overall, the CNN-BDM-based Lite-V2 architecture provides an efficient, explainable, and deployment-ready solution for automated crack and surface-type recognition in resource-constrained environments. Future work will aim to enhance fine-crack detection through domain-driven synthetic augmentation and adaptive fine-tuning for unseen domains. In addition, integrating temporal information and multimodal sensing (e.g., infrared or LiDAR data) may further strengthen robustness and generalization, paving the way for next-generation, real-time structural health monitoring systems.

## Data Availability

The datasets used and/or analysed during the current study available from the corresponding author on reasonable request.
